# Cutting-edge exploration of insect utilization in ruminant nutrition—feature and future: a systematic review and meta-analysis

**DOI:** 10.3389/fvets.2024.1484870

**Published:** 2024-11-20

**Authors:** Min Gao, Mohamed El-Sherbiny, Małgorzata Szumacher-Strabel, Adam Cieślak, Yulianri R. Yanza, Agung Irawan, Biao Xie, Zhi-jun Cao, Isa Fusaro, Hassan Jalal, Ahmed M. Abd El Tawab, Yong-bin Liu

**Affiliations:** ^1^State Key Laboratory of Reproductive Regulation and Breeding of Grassland Livestock, Inner Mongolia University, Hohhot, China; ^2^Department of Dairy Science, National Research Centre, Giza, Egypt; ^3^Department of Animal Nutrition, Poznań University of Life Sciences, Poznań, Poland; ^4^Department of Animal Nutrition and Feed Technology, Faculty of Animal Husbandry, Universitas Padjadjaran, Jatinangor, West Java, Indonesia; ^5^Vocational School, Universitas Sebelas Maret, Surakarta, Indonesia; ^6^Department of Animal and Rangeland Sciences, Oregon State University, Corvallis, OR, United States; ^7^State Key Laboratory of Animal Nutrition and Feeding, College of Animal Science and Technology, China Agricultural University, Beijing, China; ^8^Department of Veterinary Medicine, Faculty of Veterinary Medicine, University of Teramo, Teramo, Italy; ^9^CAS Key Laboratory for Agro-ecological Processes in Subtropical Region, National Engineering Laboratory for Pollution Control and Waste Utilization in Livestock and Poultry Production, Hunan Provincial Key Laboratory of Animal Nutritional Physiology and Metabolic Process, Institute of Subtropical Agriculture, Chinese Academy of Sciences, Changsha, Hunan, China

**Keywords:** insects as feed, *in vitro* digestibility, *in vivo*, methane, total gas production

## Abstract

There has been a growing interest in using insects as sustainable protein sources for ruminant feed, such as the adults of the two-spotted cricket (*Gryllus bimaculatus*), larvae of the mealworm beetle (*Tenebrio molitor*), black soldier fly (*Hermetia illucens*), and pupae of the silkworm (*Bombyx mori*). The advantages of these insects over other plant materials lie in their elevated levels of crude protein and fat. However, this interest lacks a comprehensive understanding of the impact of insects on the ruminal fermentation processes, including digestibility and gas production, as well as the impact on animal performance and related health aspects. This review offers a comprehensive analysis of ruminal fermentation indices across diverse insect species. Employing descriptive and meta-analysis methodologies, we examined the impact of incorporating insect-derived meals in ruminants’ diets. Moreover, we evaluated the growth performance and biochemical parameters of blood in ruminants when species such as *Tenebrio molitor*, *Hermetia illucens*, Oriental Hornet (*Vespa Orientalis*), and *Bombyx mori* were incorporated into ruminants’ diets. The meta-analysis was performed on a limited dataset of 14 *in vitro* and eight *in vivo* trials, investigating insect meal as a potential feed source. A comparison is drawn between these insect-based feeds and conventional dietary sources such as soybean meal, alfalfa hay, and commercial concentrate diets. Our meta-analysis revealed that incorporating *Gryllus bimaculatus* and *Hermetia illucens* to partially replace protein sources in ruminants’ diet did not adversely affect digestibility, ruminal fermentation, and ruminant production, supporting the feasibility as a feed ingredient for ruminant animals. In addition, the oriental hornet showed an overall higher outcome on the final BW, ADG, digestibility, and volatile fatty acid (VFA) production, suggesting the promising effect of this insect for future use in ruminants. The data also indicates that dietary insect inclusion levels should not exceed 30% (DM basis) to achieve an optimal ruminal fermentation profile. Furthermore, it offers comparative insights into the nutritional value of these insects, which warrant further investigation at the *in vivo* level. Ultimately, the existing understanding of the nutritional utilization potential of these insects by ruminants, particularly concerning macro- and micronutrients, is evaluated and revealed to be significantly constrained.

## 1 Introduction

By the year 2050, the global human population is projected to reach approximately 9.5 billion, necessitating a corresponding 70% increase in demand for animal-based food production, such as milk and meat (1). The primary livestock categories include pigs, with a production of 112.33 million metric tons (MT); poultry, with 109.02 million MT; and cattle (including beef and buffalo meat), with 67.99 million MT, collectively representing 91.80% of global meat production (2). As the most populous country globally, China has a significant demand for livestock products, particularly those derived from ruminant animals. Consequently, this surge in demand for animal-derived products may escalate the need for livestock feed (3). Providing sufficient feed for livestock is anticipated to encounter challenges as available land for cultivating feed resources diminishes. Intensive livestock production systems heavily depend on soybean meal (SBM) as a primary source of protein and essential amino acids (4). Nevertheless, its extensive use raises concerns regarding environmental sustainability and its competition with human nutrition (5).

Insects represent promising and innovative feed ingredients due to their valuable chemical composition. They are notably rich in proteins and contain significant amounts of lipids, making them suitable as protein and energy sources in animal dietary formulations (6). Insects have been incorporated as a feed ingredient in various animal species, including broiler chickens (7), laying hens (8), turkey (9), ducks (10), quail (11), rabbit (12), swine (13), companion animals (14), and aquatic species (15, 16). Despite their widespread use across these species, the utilization of insects in ruminant diets has been relatively limited. The limited adoption of insects in ruminant diets may be attributed to concerns regarding the potential risk of transmitting bovine spongiform encephalopathy (mad cow disease) despite the absence of evidence supporting such a linkage to date (17). While ruminants primarily consume grasses, legumes, and agricultural by-products, they frequently require protein supplements to enhance their production efficiency (18).

Moreover, feed expenses represent a significant constraint on advancing the livestock production industry. Feed costs typically constitute around 50–70% of the total budget, with protein requirements alone accounting for more than 15% of the overall feed expenditure (19). Hence, to address the rising demand for animal products in the coming years, there is an urgent need for innovative solutions and sustainable alternatives to traditional protein sources in animal diets, aiming to minimize environmental impact. The utilization of insects as animal feed offers substantial environmental benefits compared to conventional sources. Insects play a key role in bioconverting waste materials, require less water and land for cultivation, and contribute to lower greenhouse gas (GHG) emissions (5). Therefore, this development has prompted policymakers in the European Union to approve the use of insects as feed for pigs and poultry in April 2021, in addition to their existing approval for aquaculture since July 2017 (20). Currently, there is limited available data on the utilization and effects of insects as alternative feed for ruminants. Therefore, further scientific research is needed to raise awareness of this topic among policymakers in Europe, China, and globally and to assist in establishing a regulatory framework for licensing insects as ruminant feed (21).

The utilization of insects as feed for monogastric animals has been extensively reviewed (22). However, there remains a gap in applying insects as feed for ruminants. In Asia, Africa, Oceania, and South America, insects have historically served as traditional food sources within these regions. They have recently garnered interest as alternative protein sources in additional regions, including Europe and North America (23). In the United States, the black soldier fly (*Hermetia illucens*) is utilized exclusively in aquaculture. In Canada, the use of *Hermetia illucens larvae* is approved for both aquaculture and poultry production. Brazil currently lacks specific legislation addressing this matter, with insects permitted only for feeding non-ruminant animals (24). In countries like China and South Korea, this matter has no restrictions or limitations (25).

**Figure 1:**
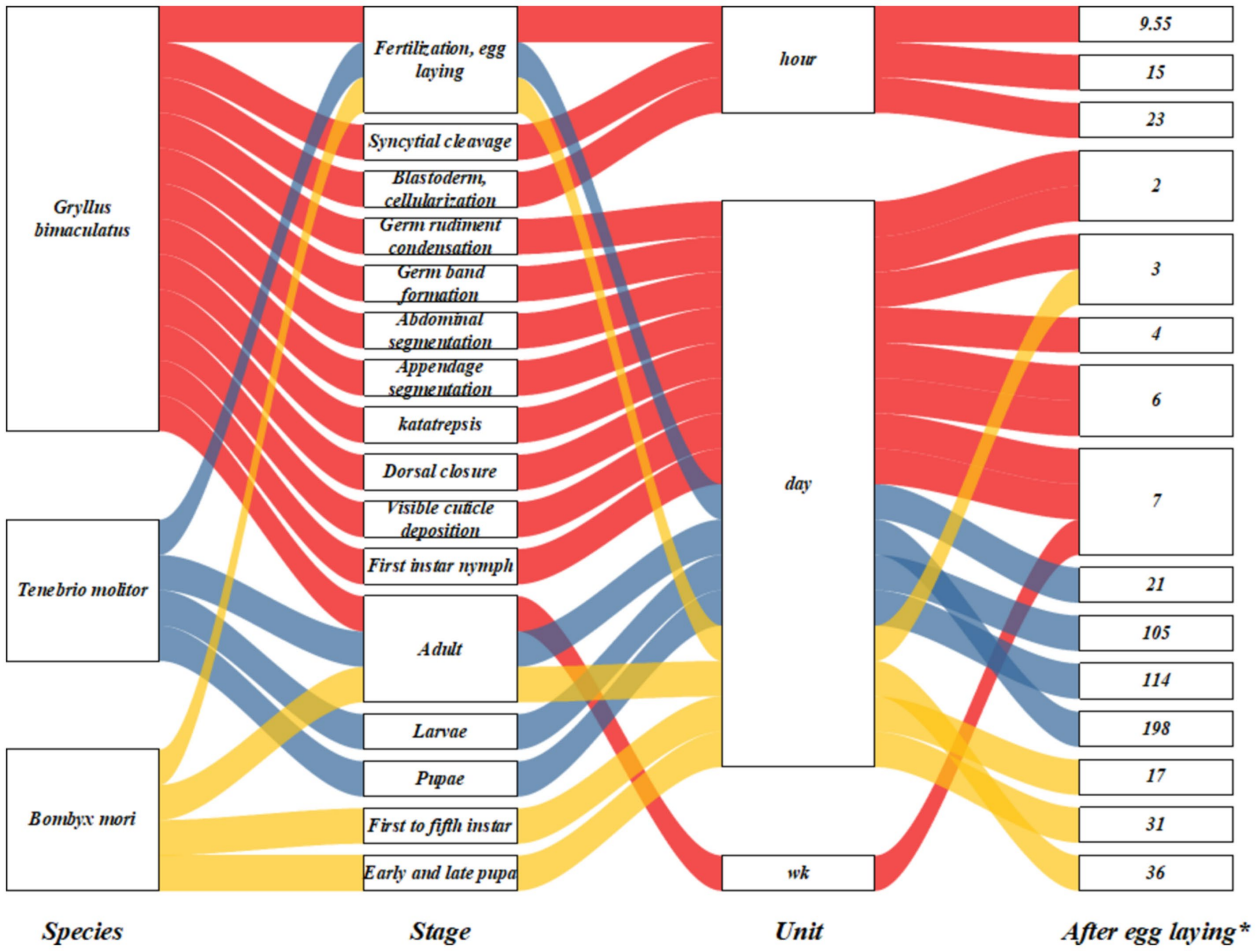
Schematic processes indicating a complete life cycle throughout major developmental *Gryllus bimaculatus*, *Tenebrio molitor*, *and Bombyx mori*; wk., week; *up to value, for example, in *Gryllus bimaculatus*, the egg-laying stage lasts from 0 to 9.55 h, etc.

Presently, there is a lack of statistical data regarding the commercial rearing of insects. However, numerous countries have begun farming insects like crickets for the feed market. Annual insect meal production is anticipated to increase to 1.2 million tons by 2025 (26). Cricket presents promising potential as an alternative feed resource for animals. Figure 1 illustrates the detailed life cycle of selected insect species in this context. Typically, crickets are reared for approximately 5–6 reproductive cycles before being discarded due to diminished productivity. These discarded crickets can subsequently be utilized as animal feed (27). The black soldier fly (*Hermetia illucens*) exhibits a life cycle lasting approximately 40–43 days (28). Research interest in using edible insects as alternative feed has surged recently. As Hanönü et al. (29) reported, insect farming emerges as a more cost-effective option when evaluating land allocation for forage crop cultivation and their associated water requirements. Insects exhibit efficient feed conversion rates and rapid growth rates. It is estimated that approximately 2 kg of organic waste and 1 m^2^ of space could yield 1 kg of insect protein. In particular, *Hermetia illucens* has gained increasing commercial utilization in animal feed due to its ease of rearing, high productivity, rich nutritional content, and efficient organic waste utilization. *Hermetia illucens larvae* have demonstrated the capacity to consume substrate ranging from 25 mg to 500 mg of fresh matter per larva per day, achieving a body length of approximately 27 mm, width of 6 mm, and weight of 220 mg by 14 days of age (30).

**Figure 2:**
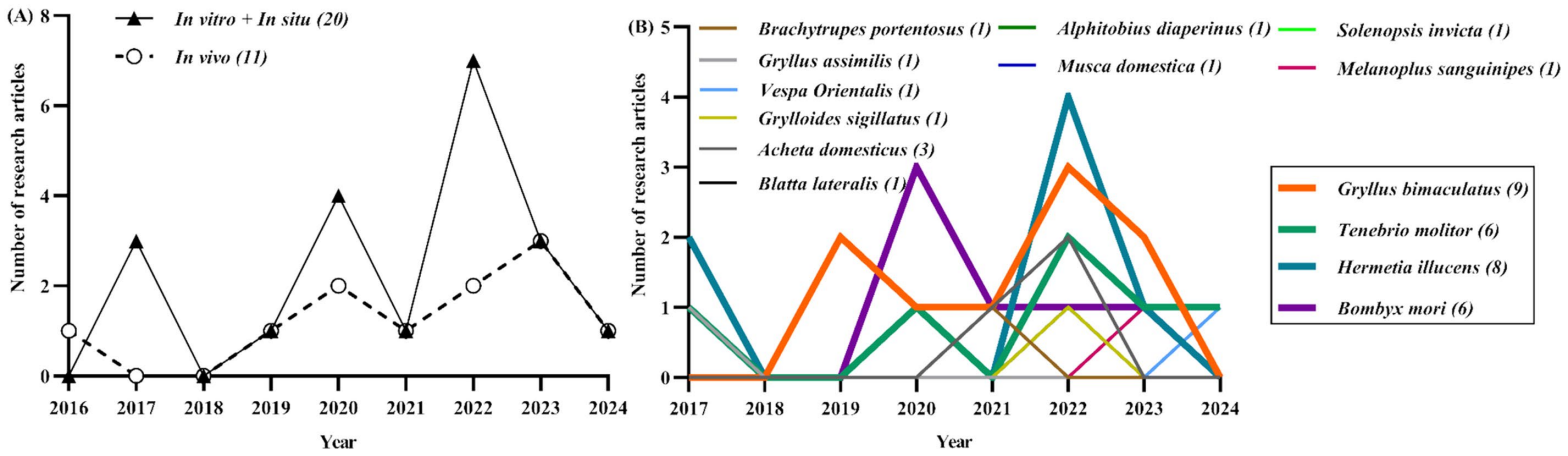
The number of original scientific research articles (excluding review articles) specifically investigating the relationship between insects and ruminant nutrition was determined using Google Scholar, Science Direct, PubMed, Web of Science, and Scopus databases. The search utilized the following keywords: **(A)** “insect, rumen” or **(B)** the names of insect species previously studied concerning ruminants by researchers. The highlighted insect species (bolded lines—*Gryllus bimaculatus*, *Tenebrio molitor*, *Hermetia illucens*, and *Bombyx mori*) were emphasized due to their frequent study in this context.

Moreover, Indonesia, characterized by its archipelagic geography and tropical climate, offers favorable conditions for *Hermetia illucens* production. Data from the Indonesian Ministry of Fisheries in 2021 indicate the presence of over 175 *Hermetia illucens* farmers spanning from the Sumatra Island (western) to the Papua Island (eastern) regions, with an average production rate of 100 kg per day (2). One challenge associated with small-scale production is the cost factor. Implementing good manufacturing practices could help mitigate the production costs of insects. Numerous insect species have undergone evaluation as potential components of ruminant diets, with notable candidates including the larvae of the mealworm beetle (*Tenebrio molitor*), pupae of the silkworm (*Bombyx mori*), larvae of the black soldier fly (*Hermetia illucens*), and adult two-spotted cricket (*Gryllus bimaculatus*) (Figure 2). However, research into consumer and stakeholder perspectives regarding the use of insects in farm animal diets remains limited. The chemical composition and average nutritional values of commonly studied insects used in ruminant nutrition research, such as *Gryllus bimaculatus* adults, *Tenebrio molitor* larvae, *Hermetia illucens* larvae, and *Bombyx mori* pupae are fully described in Table 1.

**Table 1:** The nutritive value variability of the selected insect species^1^.

Item	*Gryllus bimaculatus*		*Tenebrio molitor*		*Hermetia illucens*		*Bombyx mori*	
Commonly name	*Two-spotted cricket*		*Mealworm beetle*		*Black soldier fly*		*Silkworm*	
Family	*Gryllidae*		*Tenebrionidae*		*Stratiomyidae*		*Bombycidae*	
Order	*Orthoptera*		*Coleoptera*		*Diptera*		*Lepidoptera*	
Stage	*Adult*	(n)	*larvae*	(n)	*larvae*	(n)	*Pupae*	(n)
Dry matter, %	95.5 ± 1.16	4	95.4 ± 3.17	12	94.3 ± 4.24	10	93.5 ± 3.61	8
Organic matter, % DM	94.9 ± 2.25	7	95.5 ± 1.35	21	92.1 ± 3.68	16	93.7 ± 3.95	20
Crude ash, % DM	5.06 ± 2.24	7	4.47 ± 1.35	21	7.26 ± 2.78	15	6.33 ± 3.95	20
Crude protein, % DM	59.8 ± 4.95	8	51.7 ± 6.08	28	43.9 ± 8.51	17	59.7 ± 12.3	44
Crude fat, % DM	21.3 ± 11.3	8	29.7 ± 5.20	25	25.2 ± 9.98	15	23.2 ± 8.99	33
Crude fiber, % DM	8.75 ± 1.34	5	6.21 ± 1.06	8	6.00 ± 2.69	2	7.01 ± 4.20	16
Nitrogen-free extract, % DM	10.1 ± 5.13	3	14.5 ± 1.13	2	ND	-	5.44 ± 1.17	2
Neutral detergent fiber, % DM	35.4 ± 3.22	2	15.2 ± 5.90	6	22.7 ± 8.67	7	35.3 ± 7.01	2
Acid detergent fiber, % DM	18.5 ± 8.14	2	8.98 ± 1.80	9	10.5 ± 5.32	7	14.9 ± 8.10	2
Chitin, % DM	6.48 ± 0.95	2	6.59 ± 2.23	16	4.51 ± 1.29	8	8.61 ± 1.73	2
Gross Energy, kcal/100 g	456.3 ± 70.6	4	598.7 ± 66.2	9	499.3 ± 71.7	3	543.4 ± 77.9	3
Minerals, mg/100 g DM								
Macrominerals								
Calcium (Ca)	152.3 ± 77	4	29.2 ± 7.77	12	2,155 ± 879	15	97.6 ± 53.0	7
Phosphorus (P)	918 ± 235	3	926.7 ± 200	15	772.1 ± 202	14	689.4 ± 134	5
Potassium (K)	1,053 ± 38.7	2	911.5 ± 162.1	10	1,528 ± 519	16	618 ± 193	5
Sodium (Na)	334.2 ± 149.4	3	157.7 ± 69.4	8	344.2 ± 189	12	35.7 ± 8.67	2
Magnesium (Mg)	109 ± 35.3	3	227.7 ± 39.2	10	341.8 ± 135	17	211.5 ± 85.2	6
Microminerals								
Manganese (Mn)	6.97 ± 3.48	3	1.19 ± 0.41	9	18.2 ± 3.95	13	1.60 ± 0.44	6
Iron (Fe)	8.44 ± 3.32	4	5.52 ± 2.01	9	30.9 ± 8.11	12	3.55 ± 0.77	6
Copper (Cu)	3.42 ± 1.41	3	1.57 ± 0.35	10	0.84 ± 0.11	11	1.06 ± 0.31	5
Zinc (Zn)	18.6 ± 5.24	4	12.7 ± 3.04	10	10.6 ± 3.21	11	14.1 ± 6.90	5
Fatty acids, g/100 g DM (unless otherwise stated)								
C18:1 *c*9 Oleic	6.16 ± 4.59	2	14.7 ± 1.17	2	12.4 ± 5.18	18	31.1 ± 8.99^a^	5
C18:2 *c*9*c*12 Linoleic	2.77 ± 1.95	2	7.38 ± 0.27	2	17.4 ± 6.49	18	6.80 ± 2.94^a^	18
C18:3 *c*9*c*12*c*15 α-linolenic	0.05 ± 0.04	2	0.12 ± 0.01	2	1.65 ± 0.59	18	30.9 ± 10.5^a^	18
∑ n-3 FA	0.08 ± 0.003	2	0.11 ± 0.00	1	2.06 ± 0.81	18	ND	-
∑ n-6 FA	2.90 ± 1.91	2	7.67 ± 0.00	1	18.6 ± 6.12	18	ND	-
∑ SFA	8.01 ± 6.73	2	6.70 ± 0.34	2	62.5 ± 10.6^a^	18	34.5 ± 0.00^a^	1
∑ MUFA	6.49 ± 4.75	2	16.6 ± 0.00	1	15.9 ± 5.09^a^	18	57.0 ± 0.00^a^	1
∑ PUFA	3.07 ± 1.79	2	7.78 ± 0.00	1	22.9 ± 3.79^a^	12	8.50 ± 0.00^a^	1
Total FA	17.6 ± 9.69	2	29.9 ± 2.05	2	23.4 ± 8.84	10	-	-
Amino acids, % DM (unless otherwise stated)								
EAA								
Histidine	2.04 ± 0.66	2	2.11 ± 0.67	14	1.19 ± 0.22	8	9.07 ± 6.32^b^	6
Lysine	2.66 ± 0.33	2	3.07 ± 0.84	14	2.22 ± 0.47	8	5.33 ± 2.23^b^	7
Threonine	1.84 ± 0.23	2	2.34 ± 0.56	14	1.60 ± 0.47	8	5.68 ± 2.43^b^	7
Isoleucine	2.26 ± 0.13	2	2.28 ± 0.42	14	1.68 ± 0.32	8	2.83 ± 0.95^b^	7
Leucine	3.93 ± 0.06	2	3.43 ± 0.55	14	2.51 ± 0.69	8	4.42 ± 2.31^b^	7
Methionine	0.57 ± 0.42	2	0.78 ± 0.24	13	0.79 ± 0.19	9	4.02 ± 1.74^b^	5
Phenylalanine	2.04 ± 0.29	2	2.27 ± 0.66	14	1.55 ± 0.27	8	4.08 ± 2.54^b^	7
Tryptophan	0.27 ± 0.00	1	0.36 ± 0.08	8	0.58 ± 0.05	3	1.58 ± 0.21^b^	5
Valine	3.35 ± 0.21	2	3.32 ± 0.71	14	2.32 ± 0.57	8	4.63 ± 1.04^b^	7
NEAA								
Arginine	3.54 ± 0.09	2	3.18 ± 0.71	14	1.77 ± 0.41	8	3.05 ± 1.44^b^	7
Tyrosine	2.75 ± 0.03	2	3.83 ± 0.76	13	2.63 ± 0.50	5	5.77 ± 1.27^b^	7
Cysteine	2.74 ± 0.34	2	1.04 ± 0.79	10	0.46 ± 0.38	10	0.92 ± 0.50^b^	5
Alanine	5.17 ± 0.67	2	4.27 ± 0.93	13	2.67 ± 0.81	8	5.72 ± 2.13^b^	7
Glycine	3.32 ± 0.01	2	2.64 ± 0.42	13	2.59 ± 1.09	10	6.95 ± 2.02^b^	7
Proline	2.40 ± 0.58	2	4.14 ± 1.24	13	2.43 ± 0.81	8	6.51 ± 2.68^b^	7
Glutamic	6.58 ± 0.27	2	6.45 ± 1.12	13	3.94 ± 1.56	8	16.2 ± 4.00^b^	7
Serine	2.03 ± 1.00	2	2.36 ± 0.42	13	1.69 ± 0.45	9	5.63 ± 2.97^b^	7
Aspartic	3.24 ± 0.52	2	4.47 ± 1.28	13	3.36 ± 0.93	8	8.23 ± 4.01^b^	5

1 The presented values are based on the literature listed separately in Supplementary material 3.

a Values given as a percentage of the total fatty acid and their respective SD.

b Values are given as a percentage of the total AA and their respective SD.

EAA, Essential amino acid; NEAA, Non-essential amino acid; ND, Not detected. Data are presented as the mean ± SD.

Consequently, this article is structured as follows: following the introduction, the second section discusses insects’ effects on *in vitro* ruminal fermentation characteristics, mainly focusing on ruminal digestibility and gas production *in vitro*. The third section is centered on the evidence of *in vivo* studies investigating insects on ruminants, including the impacts on ruminal fermentation, productive performance, and health. The fourth section presents an economic evaluation of insect protein compared to alternative protein sources. The fifth section then thoroughly examines the legislative framework necessary for introducing a novel protein source into the specific sector of ruminant nutrition, specifically scrutinizing the governmental regulations governing insect utilization in the European Union. The sixth section delves into ethical considerations surrounding the use of insects. The seventh section summarizes the current research gaps and outlines future directions for applying insects in ruminant nutrition. Subsequently, the eighth section (Supplementary material 1) offers insights into statistical modeling and prediction of the optimal inclusion level of insects in the ruminants’ diet based on *in vitro* and *in vivo* studies, focusing on crucial aspects of ruminal fermentation driven by data availability. Finally, the conclusion will recapitulate the existing challenges and propose avenues for future research in this domain.

## 2 Ruminal digestibility and gas kinetics affected by various insects-based feeds

### 2.1 *Gryllus bimaculatus* adults

**Figure 3:**
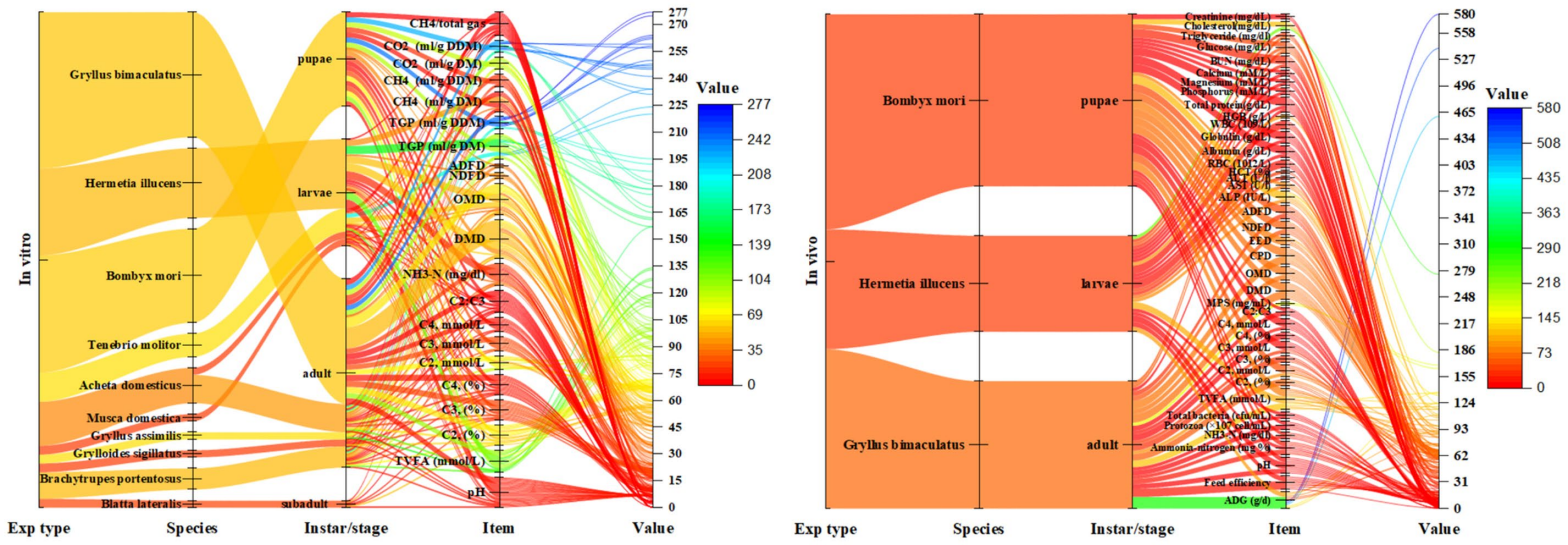
The comprehensive assessment of the impact of various insect species on ruminant nutrition, as examined through both *in vitro* and *in vivo* studies; NH_3_, Ammonia; TVFA, Total volatile fatty acids; C_2_, Acetate; C_3_, Propionate; C_4_, Butyrate; DMD, Dry matter digestibility; OMD, Organic matter digestibility; NDFD, Neutral detergent fiber digestibility; ADFD, Acid detergent fiber digestibility; TGP, Total gas production; DM, Dry matter; DDM, Degraded dry matter; CH_4_, Methane; CO_2_, Carbon dioxide. The value means of the corresponding measurements obtained from the original data gathered across all investigations encompassing insects on ruminants, as examined in this paper (both *in vitro* and *in vivo*), were depicted graphically.

Supplementary Tables S1, S2 (Supplementary material 2) presents the *in vitro* experiments examined in this review regarding the impact of different insects on ruminal fermentations. The detailed ruminal *in vitro* fermentation profiles of specific insects, *Gryllus bimaculatus* and *Bombyx mori*, have been outlined in Supplementary Table S6 (Supplementary material 2). Figure 3 illustrates the ruminal fermentation metrics based on *in vitro* and *in vivo* studies examining the effects of various insect species and morphological stages. In a study conducted by Renna et al. (5), the *in vitro* ruminal fermentation characteristics were examined after 24 h of incubation using *Gryllus bimaculatus* adult meal as the incubation substrate, compared with control meals. The findings revealed significant reductions in total gas production with *Gryllus bimaculatus* adult meal treatments, showing 72.6, 70.6, and 57.3% for soybean, rapeseed, and sunflower meals, respectively. Similarly, methane (CH_4_) production was notably lower with *Gryllus bimaculatus* adult meal treatments, with reductions of 79, 73.9, and 62.4% for meals of soybean, rapeseed, and sunflower, respectively. However, due to high fat and chitin content, the *in vitro* organic matter digestibility (IVOMD) in *Gryllus bimaculatus* adult meal treatments also decreased significantly by 45, 36, and 21% compared to soybean meal, rapeseed meal, and sunflower meal, respectively. Furthermore, the total saturated fatty acid (SFA) content was significantly lower by 6.79% compared to the soybean meal group. Nonetheless, the digestibility of *Gryllus bimaculatus* adult meal was relatively low, potentially limiting their utilization as feed ingredients.

To enhance the feeding value of *Gryllus bimaculatus* adult meals, specific treatments or processing methods targeting the removal of the exoskeleton fraction or chitin may be necessary. Comparative analysis of these findings with other studies demonstrates the impact of cricket exoskeleton removal through manual methods (removing the head, legs, and wings of oven-dried crickets) or chemical extraction (delipidation), resulting in a chitin reduction of 54.5 and 100%, respectively. When used as the sole substrate for *in vitro* ruminal fermentation, exoskeleton removal and chemical extraction of crickets were found to increase *in vitro* dry matter digestibility—IVDMD (and IVOMD) by 1.9% (2%) and 2% (1.7%) compared to whole cricket meal, respectively. Furthermore, in the same study, researchers reported that using crickets after exoskeleton removal and whole cricket meal to fully replace SBM at an inclusion level of 30% in the diet increased IVDMD by 1.9% and IVOMD by 2.7%, respectively. However, these increases were not statistically significant (27). In a study conducted by Ahmed et al. (19), it was observed that supplementing the diet with 10% *Gryllus bimaculatus* adult meal resulted in the replacement of 25% of SBM in the control group. This substitution significantly reduced total gas production by 16.5 and 12.1% per gram of DM and digestible dry matter (DDM), respectively. CH_4_ production also decreased significantly by 26.7 and 22.5%, respectively. Interestingly, the digestibility of DM, OM, NDF, and ADF did not exhibit significant differences and yielded similar results between the experimental and control groups. These findings suggest that the investigated insects could be a sustainable alternative to replace 25% of the high-quality and expensive protein source, soybean meal, at a 10% inclusion level without inducing any adverse effects.

These findings highlight the potential of using *Gryllus bimaculatus* adult meal as a viable and environmentally friendly protein source in livestock feed formulations. Further research is warranted to explore long-term effects on animal performance and health. The optimal inclusion levels of *Gryllus bimaculatus* adult’s meal in ruminant diets have been demonstrated to play a pivotal role. In a recent study by Khonkhaeng et al. (31), the inclusion of *Gryllus bimaculatus* adult meal in ruminant diets was examined across a range from 65.1% to 70% by eight treatments, with a gradient increase of 0.7% for each treatment. The authors observed that when *Gryllus bimaculatus* adult meals were included at levels up to 67.9% in the diet, there was a significant linear decrease in IVOMD without affecting total gas production. However, concerning IVDMD, it was suggested that *Gryllus bimaculatus* adult meal in ruminant diets should be maintained at levels below 65.1% to avoid compromising IVDMD. This outcome is contrary to that of Ahmed and Nishida (1), who observed a linear decrease in IVDMD with the inclusion of *Gryllus bimaculatus* adults at 30% of the diet compared to the control group; this study aligns with existing literature. Specifically, it observed reductions of 16.6% in total gas production and 12.5% in CH_4_ production, consistent with prior research. Hence, the authors concluded that including *Gryllus bimaculatus* adult meal at up to 20% of the diet did not adversely affect nutrient digestibility.

To better understand the ideal inclusion levels, future trials could explore varying forage-to-concentrate (F:C) ratios, as investigated in studies such as Khonkhaeng et al. (31) (F:C 70:30) and Ahmed and Nishida (1) (F:C 60:40). These additional investigations would contribute valuable insights into optimizing *Gryllus bimaculatus* adult meal inclusion in ruminant diets. In a previous study emphasizing the significance of F:C ratios, researchers investigated the substitution of *Gryllus bimaculatus* adult meal for SBM at varying levels (25, 50, 75, and 100% replacement) that corresponded to inclusion levels of *Gryllus bimaculatus* adult meal in the diet at 4, 8, 12, and 16%, respectively. The F:C ratios were expressly set at 60:40 and 40:60. The study revealed that IVDMD was significantly higher with a F:C ratio of 40:60 compared to the corresponding ratio of 60:40. Similarly, a reduction in CH_4_ production was observed when the F:C ratio was decreased while maintaining the same level of *Gryllus bimaculatus* adult meal in the diet (23). These findings underscore the importance of F:C ratios in optimizing nutrient utilization and CH_4_ emissions in ruminant diets supplemented with *Gryllus bimaculatus* adult meal. In summary, these findings underscore the need for further investigation, including feeding trials *in vivo*, to better understand and optimize the utilization of *Gryllus bimaculatus* adult meal as a potential feed resource.

### 2.2 *Tenebrio molitor* larvae

Regarding the impact of *Tenebrio molitor larvae* meal on ruminal fermentation characteristics, this study revealed notable effects when used as the sole substrate. *Tenebrio molitor larvae* meal significantly reduced total gas production by 68.6, 66.2, and 51% compared to soybean, rapeseed, and sunflower meals, respectively. However, IVOMD decreased by 41, 32, and 17% compared to soybean, rapeseed, and sunflower meals, respectively. Additionally, the SFA content in the ruminal fluid was reduced by 53.2, 44, and 41.1% when compared to soybean (SBM), rapeseed, and sunflower meals, respectively. These outcomes are attributed to the higher fat content in *Tenebrio molitor larvae* meal (39.2%) compared to SBM (0.6%), rapeseed meal (2.8%), and sunflower meal (1.7%) (5). This discovery aligns with the findings of Jayanegara et al. (3), who reported that the higher fat content in *Tenebrio molitor* meal (20.3%) compared to SBM (2.7%) led to a significant reduction in IVDMD and IVOMD by 29.2 and 26.1%, respectively. Moreover, total gas and CH_4_ production were significantly decreased by 46.7 and 55.1%, respectively, when *Tenebrio molitor* meal was used as the sole substrate during 24-h anaerobic *in vitro* fermentation. A noteworthy discovery emerged when comparing *Tenebrio molitor* with two other non-plant protein sources: grasshopper meal (*Melanoplus sanguinipes*) and ant egg meal (*Solenopsis invicta*). This study observed that *in vitro* ruminal fermentation decreased total gas and CH_4_ production with these alternative protein sources while maintaining IVDMD (32). The research conducted by Hanönü et al. (29) demonstrated that supplementing alfalfa hay with *Tenebrio molitor larvae* meal at levels of 0.5, 1, and 1.5% led to a significant increase in IVOMD, both linearly and quadratically. A possible explanation for this effect could be attributed to the *in situ* ruminal dry matter (DM) digestibility of *Tenebrio molitor larvae* meal, which was determined to be 85.7% after 24 h, surpassing alfalfa. The *in vitro* degradable protein content was similar (around 60%) between SBM and *Tenebrio molitor larvae* meal (33).

### 2.3 *Hermetia illucens* larvae

The detailed ruminal *in vitro* fermentation profiles of specific insects, *Hermetia illucens* and *Acheta domesticus*, have been compiled in Supplementary Table S6 (Supplementary material 2). Regarding the impact of *Hermetia illucens larvae* meal on ruminant nutrition, studies have reported a significant decrease in both IVDMD and IVOMD by 35 and 34%, respectively, when used as the sole substrate compared to SBM after a 48-h incubation period. Additionally, total gas and CH_4_ production were markedly reduced by 56.3 and 67.5%, respectively, in the *Hermetia illucens larvae* meal group compared to SBM (3). This finding broadly supports the work of other studies in this area linking *Hermetia illucens larvae* meal with IVOMD. According to Renna et al. (5), IVOMD was notably lower by 46, 37, and 22% in *Hermetia illucens larvae* meal compared to SBM, rapeseed meal, and sunflower meal, respectively. Consequently, there was a reduction in total gas production by 71.3, 69.2, and 55.3%, and CH_4_ production by 77.5, 72.1, and 59.8% by *Hermetia illucens* larvae compared to SBM, rapeseed meal, and sunflower meal, respectively. This effect is attributed to the high fat (26.9%) and chitin content (5.2%) in *Hermetia illucens* larvae. Consistent with these findings, a prior study has shown that chemically defatted (using a hexane solution) and mechanically defatted (using an expeller) *Hermetia illucens* larvae led to significant increases in IVDMD and IVOMD by 26.7% (27.1%) and 14.9% (26.5%), respectively, compared to the intact *Hermetia illucens* larvae meal group. These effects were observed when these different insect inclusion levels were at 20% in the diet without influencing CH_4_ production (34). Hence, extracting fat from *Hermetia illucens larvae* is essential for optimizing the insect’s suitability as a feed ingredient for ruminant livestock. This finding aligns with recent research demonstrating that supplementing the diet with defatted *Hermetia illucens larvae* meal at 3.2%, representing a 20% substitution for SBM, led to notable increases in *in vitro* neutral detergent fiber digestibility (IVNDFD) over a 24-h incubation period, following a linear and quadratic trend compared to the control group. Moreover, IVDMD and IVNDFD exhibited enhancements of 6.31 and 4.64%, respectively, with defatted *Hermetia illucens larvae* meal at 3.2% in the diet during a 48-h incubation period. These outcomes may be attributed to the lower inclusion rate of *Hermetia illucens larvae* meal (3.2%) without significantly impacting the fat content (3.74%) when compared to the control group’s fat content (3.19%) (24).

In summary, substituting SBM with *Hermetia illucens larvae* meal in ruminant diets often reduces nutritional quality *in vitro*. The main challenges associated with incorporating *Hermetia illucens larvae* meal include their significant chitin content, indicated by elevated levels of neutral detergent insoluble crude protein and acid detergent insoluble crude protein, as well as their high-fat content, which can adversely affect ruminal digestibility. Despite these challenges, a distinct advantage of using *Hermetia illucens larvae* meal over SBM is their lower CH_4_ emissions. Enhancing the nutritional value of *Hermetia illucens larvae* meal requires the application of specific treatments or processing methods.

### 2.4 *Bombyx mori* pupae

Ahmed et al. (19) reported that including 10% *Bombyx mori* pupae meal in the diet, replacing 25% of SBM, did not affect IVDMD, IVOMD, IVNDFD, or *in vitro* acid detergent fiber digestibility (IVADFD) compared to the control group, which included 40% SBM. However, the production of carbon dioxide (CO_2_) and CH_4_ per gram of DDM was significantly reduced by 13.7 and 19.4%, respectively, in the *Bombyx mori* pupae meal group compared to the control group. Notably, chitin is a component known to be poorly digested by animals and can contribute to lower IVDMD and IVOMD. The chitin content in the *Bombyx mori* pupae meal was measured at 9.83%. The inclusion of insects at a substitution level of 25% for SBM in this study did not negatively affect nutrient digestibility, likely due to the relatively low inclusion rate employed. Additional research is needed to explore the effects of higher inclusion levels of this insect, mainly when replacing soybean meal entirely. This investigation could assess their potential as effective options for reducing CH_4_ production in ruminants.

Therefore, Ahmed and Nishida (1) conducted a study examining the inclusion of different levels (10, 20, 30, and 40%) of *Bombyx mori pupae* meal in the diet. The authors observed that including *Bombyx mori pupae* meal up to 30% in the diet resulted in a linear and quadratic decrease in IVDMD compared to the control group, which consisted of 300 mg of grass hay and 200 mg of concentrate mixture during a 24-h fermentation period. Furthermore, including 20% *Bombyx mori pupae* meal in the diet was deemed a safe threshold as it did not significantly impact IVDMD but led to a notable reduction in total gas and CH_4_ production by 9.2 and 9.9%, respectively. It suggests that a 20% inclusion level of *Bombyx mori pupae* meal could be a suitable option for minimizing CH_4_ emissions without affecting DM digestibility. Further trials were conducted using different F:C ratios to better understand the ideal inclusion levels. Based on the findings, it can be concluded that supplementing *Bombyx mori pupae* oil at a 2% level reduces CH_4_ production by 12%–15% without negatively impacting feed fermentation. The reduction in CH_4_ may be more notable when the oil supplement is added to a high-concentrate diet (F:C; 70:30) compared to a diet with a lower concentrate ratio (F:C; 40:60), resulting in reductions of 5.28 and 4.52%, respectively, compared to the control group (no oil supplement). Thirumalaisamy et al. (35) (Supplementary Table S7 in Supplementary material 2) presents the variations in ruminal fermentation parameters observed in response to different insects during the *in vitro* experiments. *Hermetia illucens* supplementation led to a notable decrease in acetate production by 34.5% compared to the control group (*p* = 0.03). As a result, there was a pronounced reduction in the acetate:propionate (C_2_:C_3_) ratio (*p* = 0.03; Supplementary Table S7 in Supplementary material 2).

## 3 Insects-based diet in ruminant feeding: *in vivo* trials overview

Supplementary Table S8 (Supplementary material 2) presents descriptive statistics for the variables used in assessing the impact of *Hermetia illucens*, *Tenebrio molitor*, *Bombyx mori*, and *Vespa orientalis* on blood biochemical parameters. Astuti et al. (36) documented that incorporating cricket meal at a concentration of 30% within the concentrate for post-weaning Etawah crossbred goats resulted in physiological responses (rectal temperature, heart rates, and respiration rate) that fell within normal ranges. However, the experimental group exhibited 182% significantly higher crude fat intake compared to the control group. Importantly, no adverse effects on ruminal fermentation profiles were observed, and the goats in the experimental group performed comparably to those on the control ration. These findings are consistent with earlier observations indicating that incorporating 15% cricket meal (replacing 100% soybean meal) as a protein source in lamb rations does not adversely affect palatability, performance, digestibility of DM and crude protein, feed efficiency, or blood metabolite profiles (including glucose, triglycerides, and total protein).

Furthermore, utilizing 7.5% cricket meal in lamb rations has been shown to reduce CH_4_ production, as reported by the authors significantly. Therefore, replacing soybean meal with 7.5% cricket meal may be more advantageous, considering the positive impact on CH_4_ reduction (37). Another example of this is the study carried out by Phesatcha et al. (4), which demonstrated that incorporating adult cricket meal (*Gryllus bimaculatus*) at 8% of the ration resulted in a significant linear increase of 25.6% in average daily gain and 7.46% in apparent digestibility of crude protein in Thai native male beef cattle. This increase was accompanied by linearly significant rises in rumen ammonia-nitrogen (26.5%) and blood urea nitrogen (6.4%). Furthermore, total volatile fatty acids (TVFA) were linearly increased by 26.5%, predominantly due to a 4.2% higher propionic acid level compared to the control group when cricket meal was included at 12% of the ration. Their study highlighted that cricket meal had a CP content of 62.4%, higher than soybean meal (SBM), influencing the alteration in TVFA.

Consequently, the C_2_:C_3_ ratio was significantly reduced. Moreover, estimated CH_4_ emissions decreased by 20.9%, partially explained by a 35.9% decrease in protozoa when cricket meal completely replaced SBM in the ration. These findings suggest the potential benefits of cricket meal in improving cattle performance and reducing CH_4_ emissions in feed formulations. Moreover, several research studies have recently investigated the use of defatted silkworm pupae meal in ruminant nutrition. Rashmi et al. (38) conducted a study that concluded that defatted silkworm pupae meal could be safely included at a level of 4.1% in cattle concentrate mixtures (substituting soybean meal up to 30%) without adverse effects on health or performance. This finding suggests that defatted silkworm pupae meal is a promising alternative to traditional protein sources for cattle, offering both nutritional benefits and cost advantages. A notable aspect of their study is the cost-effectiveness of defatted silkworm pupae meal compared to soybean meal. The price of defatted silkworm pupae meal was found to be 51.2% lower than soybean meal when calculated on per kilogram of crude protein basis. This cost advantage further enhances the appeal of defatted silkworm pupae meals as a viable protein source for cattle feed formulations. A notable finding from the earlier-reported results highlights the effective use of silkworm pupae oil to enhance ether extract digestibility by approximately 10% and reduce enteric CH_4_ emissions by 17.5%–20.5%. These improvements were achieved without compromising nutrient intake or digestibility when oil supplementation was administered continuously (daily) or intermittently (alternate week) at a consistent level of 2% of the diet.

Furthermore, the observed reduction in CH_4_ emissions is attributed to a decrease in protozoa population. Expressly, significant decreases were noted in total protozoa (39.8%–42%) and *Isotrichidae* (40.3%–41.8%) (39). These findings align with a meta-analysis by Dai et al. (40), which demonstrated that CH_4_ emissions correlate positively with total rumen protozoa and *Isotrichidae* but not with *Ophyroscolecidae*. In summary, using silkworm pupae oil as a supplement in livestock diets shows promise for improving nutrient digestibility and reducing CH_4_ emissions through targeted modulation of rumen microbial populations. Further research could contribute valuable insights into sustainable livestock production practices.

Only one study has explored the effects of *Oriental Hornet* meal on lamb nutrition (41). The findings from this study revealed significant improvements in the digestibility of DM, organic matter (OM), crude protein, and ether extract when *Oriental Hornet* meal was included at a level of 3.42% of the ration. Specifically, digestibility increased by 2.32, 2.99, 9.74, and 1.93%, respectively. Moreover, including *Oriental Hornet* meal at this level led to notable enhancements in average body weight gain (30.9%) and growth rate (30.7%) compared to the control group. This improvement can be attributed to the higher total digestible nutrients and digestible crude protein content in the experimental ration, which were 1.56 and 1.43% higher than the control group, respectively, due to the substitution of *Oriental Hornet* meal for SBM. An intriguing finding was the significantly increased economic efficiency of 19.1% observed in the experimental group compared to the control group. This higher economic efficiency suggests a higher net return from using *Oriental Hornet* meal, making it potentially well-suited for the Egyptian market. In summary, the limited study on *Oriental Hornet* meal in lamb nutrition demonstrated promising effects on digestibility, growth performance, and economic efficiency. Further research could provide valuable insights into the potential utilization of *Oriental Hornet* meals as a cost-effective and beneficial protein source for ruminants, particularly in specific regional markets like Egypt (41).

In addition, recent research comparing the supplementation of 4% *Hermetia illucens* oil to sheep ration vs. no supplementation has shown significant increases in both TVFA and total bacteria in the ruminal fluid, with increments of up to 44.8 and 77.1%, respectively (28). The variation in total bacterial population can be attributed to several factors, including differences in rations, types of feed, timing and methods of rumen fluid collection, and feeding frequency. Rations containing easily digestible protein and carbohydrates promote bacterial growth in the rumen. In the study by Ningsih et al. (28), the experimental diets exhibited a total digestible nutrients (TDN) content up to 5% higher than the control meal. This increase likely contributed to the observed rise in the TVFA in the rumen, presumably due to the higher bacterial population resulting from the addition of black soldier fly oil supplementation. Consistent with the findings of this study, previous research has shown that the addition of *Hermetia illucens* fat at a level of 0.2% in the ration of multiple-breeding black-motley cows resulted in a significant increase in TVFA production in the rumen (42).

Recent investigations have explored the impact of incorporating *Hermetia illucens* meal into sheep nutrition. Researchers observed that replacing soybean meal with black soldier fly larvae did not negatively affect the performance or hematological profile of the sheep. Notably, body weight gain tended to increase (*p* = 0.082), and feed conversion ratio tended to decrease (*p* = 0.089) when *Hermetia illucens larvae* meal was included at 2.5 and 5% of the ration, respectively. Furthermore, analysis of blood leukocyte differentiation, including lymphocytes, monocytes, neutrophils, eosinophils, and basophils, showed no significant differences, indicating that all animals maintained a healthy status (43). Because lymphocytes play a central role in adaptive immunity, recognizing and targeting specific pathogens. Monocytes can differentiate into macrophages upon entering tissues, where they play a vital role in engulfing and digesting pathogens. Neutrophils, the most abundant white blood cells, act as the body’s primary defense against infections by engulfing and destroying bacteria through phagocytosis. Eosinophils combat parasitic infections and regulate allergic responses by releasing toxic proteins. Basophils release histamine and other chemicals involved in allergic reactions, contributing to the inflammatory response and defense against certain parasites (44).

Moreover, Supplementary Tables S1–S5 (Supplementary material 2) contains a comprehensive list of both *in vitro* and *in vivo* experiments discussed in the review, detailing experimental methodologies such as methods used, incubation times or experimental periods, information about animal donors including their status and feeding regimens, specifics of treatments applied, insect species studied, and ethical approvals obtained. Besides, depending on the species, form, and inclusion level of insects, substituting soybean meal can have varying degrees of impact on ruminal fermentation indices and performance, as detailed in Table 2. Moreover, descriptive statistics of the variables in the database used to evaluate the effect of *Gryllus bimaculatus, Hermetia illucens,* and *Bombyx mori* on ruminal fermentation parameters in ruminants (*in vivo*) have been shown in Supplementary Table S9 (Supplementary material 2). Supplementary Table S10 (Supplementary material 2) displays the impact of various insects on ruminal fermentation parameters. The *Gryllus bimaculatus* treatment yielded a significant increase (*p* < 0.01) in ruminal pH, rising by 3.76% compared to the control. *Vespa Orientalis* treatments enhanced the apparent digestibility of DM by 7% (*p* = 0.003) compared to the control.

**Table 2:** Effect of various invertebrate insects used as either protein or fat (energy) source carrier on the ruminant species response.

Source (*form*)	Species	Replaced meal	Inclusion level (*Substitution level*)	Results	Reference
*Gryllus bimaculatus*	Beef cattle^1^	Soybean meal	4%; 8%; 12%	Replacing SBM with cricket meal in the concentrated feed mixture at up to 100% improved nutrient digestibility and ruminal fermentation efficiency in Thai native beef cattle fed a diet primarily composed of rice straw. This substitution resulted in increased production of volatile fatty acids, particularly propionate, and enhanced microbial protein synthesis. Additionally, protozoal populations decreased, and CH_4_ production in the rumen was mitigated.	(4)
(*Full-fat meal pellet*)			(*33%; 67%; 100%*)		
*Hermetia illucens*	Sheep^2^	Supplement oil	4%	The addition of calcium soap black soldier fly oil to the ration of Garut sheep has been shown to elevate total volatile fatty acid levels and bacterial population without affecting rumen pH, ammonia concentration, or protozoa population.	(28)
(*Full-fat*)			(*No specific substitution*)		
*Gryllus bimaculatus*	Goats^3^	Soybean meal	15%; 30%	Incorporating cricket meal at levels of up to 30% in the concentrate portion of diets for growing goats has demonstrated favorable palatability, with no discernible adverse impacts on ruminal fermentation profiles and comparable performance relative to control rations.	(36)
(*Full-fat meal*)			(*50%; 100%*)		
*Gryllus bimaculatus*	Lambs^4^	Soybean meal	7.5%; 15%	The study findings indicate that incorporating 15% cricket meal (as a complete replacement for soybean meal) in lamb rations is feasible without compromising palatability, performance, feed efficiency, or blood metabolite profiles. Additionally, offering lamb rations with 7.5% cricket meal leads to a notable reduction in CH_4_ production. Considering these results, substituting soybean meal with 7.5% cricket meal may be more advantageous due to its CH_4_-reducing effect.	(37)
(*Full-fat meal*)			(*50%; 100%*)		
*Bombyx mori*	Steers^5^	Soybean meal	1.4%; 2.7%; 4.1%	It was determined that dried silkworm meal could be incorporated into cattle concentrate mixtures at levels of up to 4.1% as a safe substitute for SBM without adverse effects on the health or performance of the animals. Therefore, silkworm meal presents itself as a promising alternative to traditional protein sources for cattle, offering benefits in terms of both nutritional quality and cost-effectiveness.	(38)
(*Defatted*)			(*10%; 20%; 30%*)		
*Bombyx mori*	Sheep^6^	Supplement oil	2%	Silkworm pupae oil, when included at 2% of the diet, has demonstrated the capability to achieve a significant reduction of approximately 15–20% in enteric CH_4_ emissions while maintaining intake and nutrient digestibility. This reduction in CH_4_ emissions results from a combination of reduced protozoa levels and alterations in the rumen methanogen community composition.	(35)
(*Full-fat*)			(*No specific substitution*)		
*Vespa Orientalis*	Lambs^7^	Soybean meal	1.14%%; 2.28%; 3.42%	Using Oriental Hornet meal, replacing soybean meal up to 30%, can enhance productive and reproductive performance, nutrient composition, physiological responses, and economic efficiency in Ossimi lambs without detrimentally affecting their performance.	(41)
(*Full-fat meal*)			(*10%; 20%; 30%*)		
*Hermetia illucens*	Beef cattle^8^	Supplement fat	0.02%; 0.2%	The data indicates that incorporating Black Soldier Fly Larvae fat can enhance cow productivity, immune defenses, and milk quality.	(42)
(*Full-fat*)			(*No specific substitution*)		
*Hermetia illucens*	Sheep^9^	Soybean meal	2.5%; 5%	Black soldier fly larvae have the potential to replace soybean meal in sheep diets without negatively impacting performance or hematological profiles.	(43)
(*Full-fat meal*)			(*50%; 100%*)		

1 Thai native male beef cattle (2 years old; 230 ± 15 kg of BW).

2 Garut sheep (No specific statement for BW).

3 Post-weaning Etawah crossbred goat (2 months old; 12 ± 0.40 kg of BW).

4 No specific species mentioned (2 months old; 11.24 ± 1.62 kg of BW).

5 Crossbred steers (496.25 ± 5.39 kg of BW).

6 Mandya sheep (16–18 months old; 24.1 ± 1.20 kg of BW).

7 Ossimi lambs (20.58 ± 0.85 kg of BW).

8 Black-motley cows (590 ± 4 kg of BW; BCS 3.15 ± 0.04).

9 No specific species mentioned (6–8 months old; 20.42 ± 3.57 kg of BW).

BCS, Body condition score; BW, Body weight.

Furthermore, the *Bombyx mori* treatment notably increased the apparent digestibility of acid detergent fiber (ADF) compared to the control treatment (*p* = 0.007; Supplementary Table S10 in Supplementary material 2). Supplementary Table S11 (Supplementary material 2) illustrates their influence on biochemical parameters in the context of *in vivo* experiments. None of the dietary insect interventions elicited discernible alterations in the blood biochemical profiles of ruminants compared to the control.

## 4 Economic evaluation of insect protein compared to alternative protein sources

Table 3 demonstrates the economic feasibility of selected insects relative to plant-based protein sources. The current prices of soybean meal, rapeseed meal, and sunflower meal feeds are approximately €0.486, €0.3, and €0.237 per kg, respectively. Meanwhile, the current prices of *Hermetia illucens* and *Tenebrio molitor* are approximately €7.25 and €14.5 per kg, respectively. Therefore, for a comprehensive assessment between insects and traditional plant protein sources, it is essential to adjust the nutritional value based on parameters such as crude protein content or essential amino acids profile. This adjustment allows a more accurate comparison of their economic and nutritional merits. The findings indicate that replacing each euro of SBM with *Hermetia illucens* would cost 16.2 €/kg for protein, 11.9 €/kg for lysine (Lys), and 20 €/kg for methionine (Met) for farmers.

**Table 3:** The economic viability of insects in comparison to plant-based protein sources.

Potential source	*Hermetia illucens* ^1^	*Tenebrio molitor* ^1^	Soybean meal^2^^a^	Rapeseed meal^3^^a^	Sunflower meal^4^^a^
CP (%)	43.9	51.7	47.58	37.6	33.52
Lysine-L (%)	2.22	3.07	3.01	1.95	1.48
Methionine-M (%)	0.79	0.78	0.638	0.76	0.75
Sales prices (€/kg; SP)	7.25	14.5	0.486	0.3	0.237
Protein-prices (€/kg; PP)	16.5	28.0	1.021	0.798	0.707
Protein-L (€/kg; PL)	0.367	0.861	0.031	0.016	0.010
Protein-M (€/kg; PM)	0.130	0.219	0.007	0.006	0.005
PP to PP SBM*	16.2	35.2	1	1	1
PL to PL SBM*	11.9	55.3	1	1	1
PM to PM SBM*	20.0	36.1	1	1	1

1 The sales prices for *Hermetia illucens* were obtained from the European Union market, particularly Germany. For Tenebrio molitor, the sales price data originated from the European Union market, specifically the Netherlands, as Niyonsaba et al. (46) indicated.

2 The chemical composition data for SBM was adopted from Lagos and Stein (51). The average calculation was based on SBM chemical composition from five countries: China, Argentina, Brazil, the United States, and India.

3 The chemical composition data for rapeseed meal was derived from the study by Cheng et al. (52).

4 The chemical composition of sunflower meal was extracted from the research conducted by Liu et al. (53).

a The price data were obtained from the website (54) https://teseo.clal.it/en/?section=oilseeds-price-eu, accessed on April 24, 2024. The price of SBM was calculated as an average from markets in Germany, the Netherlands, Poland, Romania, and Spain. The price of rapeseed meal was calculated as an average from markets in Belgium, the Czech Republic, Denmark, Germany, Hungary, Lithuania, the Netherlands, Poland, and Romania. Similarly, sunflower meal prices were determined as an average from markets in Hungary, the Netherlands, Romania, and Spain.

*The analysis involved determining how much insect protein source would need to be allocated to match the cost of each euro of plant protein source.

Similarly, replacing each euro of SBM with *Tenebrio molitor* would lead to costs of 35.2 €/kg for protein, 55.3 €/kg for Lys, and 36.1 €/kg for Met for farmers. The elevated cost of insect meal currently limits its application in ruminant diets. Nevertheless, to be competitive, expanding the scale of insect breeding operations within companies is expected to enhance efficiency and decrease the overall cost of insect protein production over time (45). Achieving mass production remains a distant prospect. While definitive conclusions on cost reduction or profit increase in insect production were not drawn, it has been proposed that greater mechanization could lead to reduced labor costs, and utilizing low-value feed substrates may decrease operational expenses. In terms of farm output sales, commercializing insect frass as fertilizer could offer an additional income stream for insect farmers (46). The potential of insects as a viable alternative feed component is attributed to their short life cycle.

Furthermore, projections from the International Platform of Insects for Food and Feed suggest a significant rise in the utilization of insects for food and feed within the European Union. The insect volume is expected to escalate from 500 tons in 2020 to surpass 1 million tons by 2025, reaching an estimated 3 million tons by 2030, encompassing both larvae and adult forms. This upward trajectory in market demand likely mirrors the lucrative opportunities available to stakeholders engaged in insect production. This growth is anticipated to contribute to heightened consumer awareness regarding the detrimental impacts of conventional animal feed production (45).

## 5 Review of regulations governing the use of insects as feed for ruminants

Insect meals are categorized as processed animal proteins and are subject to prohibitions on their utilization in numerous high-income nations (e.g., European countries). On the contrary, developing and emerging regions often lack specific legislation. For instance, in Asia, Thailand, a leading producer of crickets, is actively developing the first set of guidelines for insect breeding. In China, insects are widely used as feed and food components in various regions, yet they have not yet been officially recognized under food law (45). In the Americas, there is no specific prohibition or approval concerning the use of insect proteins in the processing, marketing, or incorporation into animal feed within this region. In the recent past, within the European Union, the approval for incorporating insects into farm animal feed was restricted to seven specific insect species, as outlined in Commission Regulation (EU) 2017/893 Commission Regulation-EU (47). These approved species encompassed two mealworm species (*Tenebrio molitor*, *Alphitobius diaperinus*), two fly species (*Hermetia illucens*, *Musca domestica*), and three cricket species (*Acheta domesticus*, *Gryllodes sigillatus*, *Gryllus assimilis*). Over time, there has been a growing expansion in the utilization of insect species for animal feed. Domestic silkworms, which exclusively consume mulberry leaves, pose no risk of contamination from animal-origin food sources that are not permitted for insect feed. Silkworms (*Bombyx mori*) have recently been added to the roster of authorized insect species for manufacturing processed animal protein utilized in animal feed, as delineated in Commission Regulation (EU) 2021/1925 (Commission Regulation-EU) (48).

Although legal regulations regarding the use of insects as feed vary regionally, researchers and feed manufacturers have a notable global interest in promoting innovation and research in this field. In the coming years, this interest may lead to legislative changes similar to those observed for monogastric animals, facilitating broader acceptance and utilization of insects in ruminant feeding practices worldwide. In summary, regarding the current global legislative framework concerning the use of insects as feed for ruminants, both insect oil and meal are explicitly authorized in countries including Mexico, Colombia, Brazil, Morocco, Algeria, Niger, Nigeria, Sudan, South Africa, Namibia, Ethiopia, India, Australia, and New Zealand. Insect oils are authorized but not insect meals in countries such as Russia, Finland, Sweden, Norway, Iceland, the United Kingdom, Denmark, Belarus, Estonia, Ireland, France, Spain, Italy, Romania, Ukraine, and Poland. Some countries like Egypt, Ecuador, Chile, Canada, and Alaska (United States) lack specific insect regulatory frameworks. However, countries such as Argentina, Iran, Japan, North Korea, and Tunisia do not authorize insect oils or meals to be used as feed for ruminants (20). Moreover, a structured compilation of legislative documents from the European Parliament and the Council (EC) concerning insect production for food and feed is presented in Supplementary Table S12 (Supplementary material 2), arranged chronologically.

## 6 Ethical considerations for insects

Insects possess the potential to be incorporated into livestock production systems as a source of feed. However, insects must be cultivated on a large scale within a “mini-livestock” framework to be effective as feed. Because these large-scale rearing systems are relatively novel, formal industry standards and welfare regulations have not been fully established, resulting in unresolved questions related to insect welfare. Considering the significance of consumer attitudes in shaping the social acceptance of insect production, it is essential to analyze consumers’ ethical perspectives on using insects as livestock feed. As per Fukuda et al. (49), sampling involved convenience sampling of 361 adult consumers in the United States. When queried about using insects as livestock feed, 34% of respondents expressed support, 52% remained neutral, and 15% voiced opposition. Among those opposed, 58% cited ethical concerns as their rationale for opposition. Among respondents who expressed support or neutrality regarding using insects as livestock feed, 29% identified concerns related to livestock welfare, while 26% identified concerns related to insect welfare as perceived risks. These observations suggest that insect producers have an incentive to implement best practices that are perceived as fostering high-welfare conditions for their “mini-livestock” when used for livestock feed. Moreover, the findings indicate that, although the existing research on consumer acceptance is limited, it is unlikely to impede the development of the insect protein industry for feed. Nonetheless, additional research is needed to investigate consumer willingness to pay for animal products derived from animals fed with insects and assess whether insects contribute to improved acceptability, both in terms of general perception and sensory appeal, compared to conventional products (50).

## 7 Current research gaps and future directions in applying insects to ruminant nutrition

The following issues warrant attention: (1) Nutrient requirements and digestibility—research gap: limited comprehensive studies on the specific nutrient requirements of ruminants when fed insect-based diets, especially the insects’ CP conventional factor for proximate analysis not unified yet. Because a portion of the nitrogen is contained within chitin, it is also extracted during protein analysis using the traditional Kjeldahl method, resulting in overestimating the actual CP content. Future direction: to standardize the conventional factor for CP content in potential insect feeds for ruminants across various species and morphological stages of the insects. (2) Feed formulation optimization—research gap: insufficient knowledge about optimal feed formulations incorporating insect meals for different classes of ruminants (e.g., lactating cows, growing calves). Future direction: explore novel feed formulation strategies that maximize the nutritional value of insect-based feeds while ensuring balanced diets for ruminant health and performance. Investigate the synergistic effects of combining insects with other feed ingredients. (3) Long-term effects on animal health and performance—research gap: limited understanding of the long-term impact of insect-based diets on ruminant health, productivity, and reproductive performance. Future direction: conduct longitudinal studies to assess the effects of sustained insect feeding on rumen health, metabolic function, immunity, and overall animal performance over extended periods. Investigate potential benefits or challenges associated with prolonged insect-based feeding. More studies are required to understand the impact of insect-based diets on ruminal fermentation dynamics, microbial populations, and metabolite production. Investigating potential health risks or safety concerns associated with feeding insects to ruminants is essential. Studies should focus on assessing antinutritional factors, toxins, or allergens in insect-based feeds. (4) Environmental impact and sustainability—research gap: incomplete evaluation of the environmental sustainability aspects of using insects as feed in dairy production systems. Future direction: quantify greenhouse gas emissions, resource utilization, and ecological footprints associated with insect farming and incorporation into ruminant diets. Explore integrated systems that leverage insect farming for waste management and circular economy principles. (5) Consumer acceptance and market dynamics—research gap: limited understanding of consumer perceptions and acceptance of dairy products derived from ruminants-fed insect-based diets. Future direction: investigate consumer attitudes toward insect-fed dairy products, addressing concerns related to food safety, quality, and ethical considerations. Develop strategies to enhance market acceptance and promote the adoption of insect-derived feed in dairy production systems. (6) Regulatory framework and policy development—research gap: inadequate regulatory guidelines and policy frameworks governing the use of insects in ruminant nutrition. Future direction: collaborate with regulatory bodies to establish evidence-based standards for insect-derived feed safety and quality assurance. Advocate for policy changes that support the sustainable integration of insects into ruminant diets. (7) Innovative approaches and technology—research gap: limited exploration of innovative technologies and processing methods for optimizing insect-derived feed production and utilization in dairy systems. Future direction: explore novel approaches such as precision feeding, genetic selection for enhanced utilization of insect proteins, and advanced processing techniques to improve the efficiency and efficacy of insect-based ruminant nutrition. Research should explore different insect species and their processing methods to optimize nutrient bioavailability and ensure feed safety. Comparative studies between fresh, dried, and processed insects can provide valuable insights. Addressing these research gaps and advancing future directions will facilitate the broader adoption of insect-derived feed in ruminant nutrition, promoting sustainability, efficiency, and resilience in dairy production systems; especially for neonatal calves, particularly those with underdeveloped rumens, the abomasum assumes paramount importance. The abomasum comprises 60–70% of the calf’s stomach capacity and secretes gastric juices rich in hydrochloric acid and digestive enzymes. These enzymes facilitate the breakdown of proteins, fats, and carbohydrates in the ingested feed, whether insects or other nutrients, into simpler forms readily absorbed by the calf’s body.

## 8 Conclusion

Recent data confirm the feasibility of integrating insects into ruminant diets, showing predominantly positive effects on growth performance, ruminal fermentation indices, and methane mitigation. However, the absence of global uniformity in insect products highlights the need for attention and standardization. To optimize the efficiency of insect and ruminant production, comprehensive assessments of economically viable insect species should be prioritized in future studies. Moreover, (1) environmental sustainability: using insects as feed aligns with sustainability goals by reducing reliance on conventional protein sources like soybean meal, which are resource-intensive and contribute to environmental degradation. Insects have a lower ecological footprint and can be produced using organic waste streams. (2) Improved feed efficiency: Insect-derived feeds offer opportunities to optimize feed efficiency in ruminants, potentially enhancing animal performance and productivity; nevertheless, the expenses associated with feed must be tackled. (3) Consumer acceptance and market trends: despite initial consumer reservations, there is growing interest in insect-fed dairy products due to their sustainability credentials and nutritional benefits. Dairy producers can leverage this trend to diversify product offerings and capture niche markets. (4) Research and development: continued research is needed to address knowledge gaps related to nutrient requirements, feed formulation, long-term health effects, and market dynamics surrounding insect-based ruminant nutrition. (5) Policy and regulatory considerations: policymakers and industry stakeholders should collaborate to establish clear guidelines and regulations governing the use of insects in ruminant diets, ensuring food safety and quality standards are met. Adopting insect-based feed strategies holds significant promise for enhancing ruminant nutrition and advancing environmental sustainability in dairy production. Dairy producers can benefit from diversifying feed sources, reducing reliance on traditional protein sources, and improving overall feed efficiency. Researchers should prioritize studies to optimize insect-derived feed formulations and assess their long-term impacts on ruminant health and performance. Policymakers and industry stakeholders play a crucial role in facilitating the adoption of insect-based feed by establishing supportive regulatory frameworks and promoting consumer acceptance. By embracing insect-based nutrition, the dairy industry can contribute to a more sustainable and resilient agricultural future.

## Data Availability

The original contributions presented in the study are included in the article/Supplementary material, further inquiries can be directed to the corresponding authors.

## Supplementary material

SUPPLEMENTARY MATERIAL 1 Data processing and meta-analysis.

SUPPLEMENTARY MATERIAL 2 Datasheet.

SUPPLEMENTARY MATERIAL 3 References.
