# Supplementary material for: Cutting-edge exploration of insect utilization in ruminant nutrition—feature and future: a systematic review and meta-analysis
Source: Front Vet Sci. 2024 Nov 20;11:1484870. doi: 10.3389/fvets.2024.1484870 (PMC11616318; doi:10.3389/fvets.2024.1484870)
Supplement: SUPPLEMENTARY MATERIAL 1 — Data processing and meta-analysis. [file Supplementary_file_1.docx]

**Data processing and meta-analysis**

Figures 1 and 3 were generated using OriginPro software (Version 2021, OriginLab Corporation, Northampton, MA, USA), while Figure 2 was created using GraphPad Prism 9 software (Version 9, GraphPad Software, San Diego, California, USA). A flowchart of article selection based on the PRISMA protocol is presented in Figure 1. Based on the available sample size, the collected data underwent statistical analysis utilizing a mixed-model approach described by Yanza et al. (1). The analysis used the PROC MIXED procedure within SAS 9.4 software (University Edition, online). In this analysis, the studies were treated as random effects, while the concentrations of insect supplements (in vitro) or daily insect dose (in vivo) were treated as fixed effects. Statistical modeling was applied to assess continuous variables, with the concentrations or dose of insect supplementation as continuous predictor variables. The specific statistical model used was represented by equation 1:

$Y_{ij}$ = $\beta_{0}$ + $\beta_{1}X_{ij}$ + $\beta_{2}X_{ij}^{2}$+ $\beta_{3}X_{ij}^{3}$+ $S_{i}$+ $b_{i}X_{ij}$ + $e_{ij}$ (1)

In the specified statistical model, where Y_ij_ represents the dependent variable for the 𝑖^𝑡ℎ^ study at the 𝑗^𝑡ℎ^ concentration or dose level of insect supplementation. β0 is the overall intercept across all studies, a fixed effect. β1 is the linear regression coefficient of Y on X, also a fixed effect. β2 is the quadratic regression coefficient of Y on X (fixed effect). β3 is the cubic regression coefficient of Y on X (fixed effect). X_ij_ denotes the value of the continuous predictor variable (insect concentration or daily intake dose) for the 𝑖^𝑡ℎ^ study at the 𝑗^𝑡ℎ^ concentration or dose level. s_i_ represents the random effect specific to each study (𝑖^𝑡ℎ^), capturing variation between studies. b_i_ signifies the random effect of each study on the regression coefficient of Y on X. e_ij_ is the unexplained residual error associated with the 𝑖^𝑡ℎ^ study at the 𝑗^𝑡ℎ^ concentration or dose level. The CLASS statement was utilized based on insect levels and the study variable, which lacked quantitative information. Meanwhile, the RANDOM statement was applied based on different studies, acknowledging the varied concentrations of insects utilized in the in vitro experiments. These models were implemented with consideration of weighted replicates within the studies, following the approach outlined by Jayanegara et al. (2). The regression equations reported were established following the Toral et al. (3). The Root Mean Square Error (RMSE) and the adjusted R-square value for linear, quadratic, and cubic regression models use the PROC REG procedure (OUTSTAT option to output statistics including RMSE and the adjusted R-square value). Comparison of the efficacy among different insect species was conducted utilizing the subsequent statistical model for discrete predictor variables (insect species; Equation 2):

$Y_{ij}$ = $\mu$ + $S_{i}$ +$\tau_{j}$+ ${S\tau}_{ij}$ + $e_{ij}$ (2)

In this model, $Y_{ij}$represents the dependent variable, 𝜇 denotes the overall mean, $S_{i}$ stands for the random effect associated with the 𝑖^th^ study, $\tau_{j}$ signifies the fixed effect corresponding to the j^th^ level of factor τ, ${S\tau}_{ij}$ denotes the random interaction between the 𝑖^th^ study and the 𝑗^th^ level of factor 𝜏, and $e_{ij}$ represents the unexplained residual error (1). Noteworthy distinctions at *P* ≤ 0.05 or a trend at 0.05 < *P* ≤ 0.10 among the different insect species, least-square means, and Tukey's post hoc test were employed to assess the disparities among the means. Moreover, the general formula for a cubic function of one variable is represented by $f(x)={ax}^{3}+ {bx}^{2}+ cx+ d$, where 𝑎, 𝑏, 𝑐, 𝑑 are real numbers and 𝑎≠0, The maximum value of a cubic function calculation follow these steps: Firstly, compute the derivative: begin by calculating the derivative 𝑓′(𝑥) of the function 𝑓(𝑥): 𝑓′(𝑥)=${3ax}^{2}$+2𝑏𝑥+𝑐. Secondly, set the derivative to zero: set 𝑓′(𝑥)=0 and solve for 𝑥 to find critical points: ${3ax}^{2}$+2𝑏𝑥+𝑐=0. Thirdly, solve for 𝑥: use the quadratic formula to solve for 𝑥 in terms of 𝑎,𝑏, and 𝑐. The solutions will provide the 𝑥-values corresponding to critical points. Fourthly, identify the maximum point: substitute the 𝑥-value obtained from the third step into the original cubic function 𝑓(𝑥) to determine the maximum value:

𝑓($\frac{-b}{3a}$)=${a(\frac{-b}{3a})}^{3}$+${b(\frac{-b}{3a})}^{2}$+𝑐($\frac{-b}{3a}$)+𝑑 (3)

In a general quadratic model 𝑦=${ax}^{2}$+𝑏𝑥+𝑐, depending on the value of 𝑎, the function can achieve a minimum value (when 𝑎>0) or a maximum value (when 𝑎<0). The formula to determine the maximum or minimum value of the quadratic function is given by 𝑐 - $\frac{b^{2}}{4a}$. This formula calculates the 𝑥-coordinate of the vertex (or extremum point) of the quadratic function. Depending on the sign of 𝑎. The axis of symmetry of a quadratic function is a vertical line that serves as a line of symmetry, perpendicular to the 𝑥-axis and passing through the vertex of the parabola defined by the function. This axis is represented by the equation 𝑥=$\frac{-b}{2a}$, where 𝑎 and 𝑏 are coefficients of the quadratic function. The coordinates of the vertex of the quadratic function are located at ($\frac{-b}{2a}$, 𝑓($\frac{-b}{2a}$)), where 𝑓($\frac{-b}{2a}$) represents the 𝑦-value obtained by substituting 𝑥=$\frac{-b}{2a}$ into the quadratic function. This axis of symmetry provides a central reference line for the parabolic curve and facilitates the determination of key points, such as the vertex, which is critical for understanding the behavior and characteristics of the quadratic function. This meta-analysis has inherent limitations due to the limited number of available studies. Despite this constraint, the results still provide support and replenish for systematic review findings. The regression equations linking the concentration (x, %) of *Gryllus bimaculatus*, *Tenebrio molitor*, *Hermetia illucens*, and *Bombyx mori* to the measurable response (y) observed after 24 hours of in vitro incubation trials have been provided in Table SM1. Within each species, a linear relationship was observed between acetate concentration (positive slopes) and propionate production (negative slopes), as shown for *Gryllus bimaculatus* treatment (*P* = 0.003; adjusted R^2^ = 0.71 for acetate, *P* = 0.006; adjusted R^2^ = 0.90 for propionate). Similarly, for *Bombyx mori* treatment, propionate concentration (*P* = 0.005; adjusted R^2^ = 0.67) and production (*P* = 0.008; adjusted R^2^ = 0.78) also exhibited negative slopes. Propionate production exhibited a significant cubic trend at the insect level with a positive slope (*P* < 0.01) and an adjusted R^2^ of 0.61. Moreover, there was a tendency for IVOMD to demonstrate a positive slope (*P* < 0.1; adjusted R^2^ = 0.52; Table SM1). Regression equations established between a selected insect (*Gryllus bimaculatus*) intake dose (x, g) and measurable response (y) in vivo trials have been shown in Table SM2. Ammonia-nitrogen displayed a quadratic trend (*P* = 0.032), with adjusted R^2^ values reaching 0.72 (Table SM2). Furthermore, according to the assumptions of the IVOMD model, dietary insect inclusion levels should not exceed 30% to achieve an optimal in vitro organic matter digestibility of 61.7% while maintaining a ruminal fluid pH of 6.85, and the highest propionate production reached approximately 20.1 mmol/l in vitro. Regarding *Gryllus bimaculatus*, to maximize total volatile fatty acids (TVFA) production, it is recommended to maintain the fed *Gryllus bimaculatus* level at 88.8 g per animal per day, resulting in a TVFA production of 115.2 mmol/l and lowest acetate:propionate ratio (C_2_:C_3_; 2.68).

In addition, a subgroup meta-analysis was performed using “meta”, “metafor”, and “dmetar” packages in the R studio environment (RStudio version 4.3.2) (4,5). Between-study heterogeneity was assessed using Cochran’s Q statistic and *I^2^* statistic, estimated by the DerSimonian-Laird estimator (6). Insect species were considered subgroups, and random effect model (REM) analysis was fitted to estimate the subgroup effects of the study interventions vs control on the variable outcomes, following the previous approach (7). The standardized mean difference (SMD) was estimated based on Hedges' *g* effect size, weighted by the inverse-variance matrix. The results were shown in Forest Plot, whereas the SMD was presented as a 95% confidence interval (95% CI).

Our meta-analysis revealed a non-significant effect when incorporating *Gryllus bimaculatus* (SMD = 0.34; *P* > 0.05) and *Hermetia illucens* (SMD = -0.95, *P* > 0.05) to partially replace protein source in ruminants’ diet. This evidence supports the feasibility of those insects as a feed ingredient for ruminant animals, as justified above. In addition, oriental hornet feeding in small ruminants had an overall higher outcome on the final BW (SMD = 7.77, *P* < 0.01), suggesting the promising effect of this insect for future use in ruminants. The results of the subgroup meta-analysis on ADG also had a similar pattern with the final BW, in which only the oriental hornet insect had the greater effect of increasing ADG of small ruminants. In contrast, *Gryllus bimaculatus* and *Hermetia illucens* resulted in no effect. The higher palatability of oriental hornet was also observed in the higher DMI (SMD = 7.17; *P* < 0.05) when included in the diet. Those three insect species did not significantly affect feed efficiency (DMI/ADG). The oriental hornet showed a higher DMD and VFA production compared to control diets, while *Gryllus bimaculatus* and *Hermetia illucens* showed no effect on these parameters. The findings of this meta-analysis suggest that the oriental hornet insect is promising to improve ruminant productivity as it had more excellent digestibility value and produced higher VFA in the rumen as a source of energy to support the production of ruminants.

**Figure 1.** Flowchart of article selection based on PRISMA protocol.

Full-text evaluation remains pending for 60 studies

Omitted (n = 18705) due to titles with overlapping content, non-research article classification, inclusion of abstracts, and titles unrelated to the study

117 articles were imported into Mendeley for the purpose of selection, relying on an assessment of their titles and abstracts

Titles identified from Google scholar (n = 17000), Science Direct (n = 1667), PubMed (n = 70), Web of Science (n = 37), Scopus (n = 48)

Total =1026

## Eligibility

## Screening

## Identification

## Included

Abstract screening resulted in the exclusion of 57 articles

38 articles were excluded:

- The study utilized the sac incubation method to assess rumen fermentation parameters (2)
- The study has not been subject to peer review (4)
- Peste des petits ruminants study (e.g., virology study) (7)
- An exclusive focus on insect studies unrelated to ruminants (10)
- The experimental focus is limited to chitin and chitosan derived from insects, rather than using whole insects (1)
- Only the abstract, devoid of specific details (4)
- Acceptance and hedonic pricing model investigation (2)
- Fat supplementation instead of using meal (7).
- Utilizing insect frass instead of meal (1)

The quantity of studies employed for meta-analysis (in vivo: n = 8, in vitro: n = 14)

**Table SM1 Regression equations established between selected insects dose (x, %) and measurable response (y) after 24 h of in vitro incubation trials**

| **Response Parameters** | **n** | **Model** | **Parameter estimates** | | | | | | | | | | | **Adjusted R^2^** | **RMSE^b^** |
| --- | --- | --- | --- | --- | --- | --- | --- | --- | --- | --- | --- | --- | --- | --- | --- |
|  |  |  | **β0** | **SE** | **β1** | **SE** | **P value** | **β2** | **SE** | **P value** | **β3** | **SE** | **P value** |  |  |
| **Based on species** |  |  |  |  |  |  |  |  |  |  |  |  |  |  |  |
| *Gryllus bimaculatus* |  |  |  |  |  |  |  |  |  |  |  |  |  |  |  |
| pH | 5 | L | 6.736 | 0.045 | 0.001 | 0.00002 | 0.102 |  |  |  |  |  |  | 0.84 | 0.05 |
| C_2_ (g/100 g VFA) | 5 | L | 60.58 | 2.535 | 0.108 | 0.006 | 0.003 |  |  |  |  |  |  | 0.71 | 2.99 |
| C_3_ (mmol/l) | 5 | L | 24.05 | 0.707 | -0.126 | 0.01 | 0.006 |  |  |  |  |  |  | 0.90 | 0.64 |
| NH_3_ (mg/dL) | 5 | L | 14.16 | 2.577 | 0.102 | 0.099 | 0.414 |  |  |  |  |  |  | 0.72 | 1.47 |
| IVOMD (%) | 10 | C | 58.11 | 13.4 | -1.068 | 51.66 | 0.431 | 0.04022 | 0.772 | 0.181 | 0.000315 | 0.004 | 0.085 | 0.52 | 12.0 |
| CH_4_/total gas | 5 | Q | 0.092 | 0.006 | -0.0001 | 0.00006 | 0.309 | 2.35×10^-6^ | 1.23×10^-6^ | 0.307 |  |  |  | 0.98 | 0.01 |
| *Hermetia illucens* |  |  |  |  |  |  |  |  |  |  |  |  |  |  |  |
| pH | 9 | Q | 6.543 | 0.197 | 0.019 | 0.009 | 0.114 | -0.0001 | 0.00009 | 0.194 |  |  |  | 0.89 | 0.09 |
| NH_3_ (mg/dL) | 8 | L | 5.676 | 6.252 | 0.144 | 0.101 | 0.227 |  |  |  |  |  |  | 0.78 | 3.51 |
| *Bombyx mori* |  |  |  |  |  |  |  |  |  |  |  |  |  |  |  |
| C_3_ (g/100 g VFA) | 5 | L | 22.37 | 0.619 | -0.085 | 0.006 | 0.005 |  |  |  |  |  |  | 0.67 | 0.55 |
| C_3_ (mmol/l) | 5 | L | 24.55 | 1.299 | -0.128 | 0.011 | 0.008 |  |  |  |  |  |  | 0.78 | 1.15 |
| Based on insects |  |  |  |  |  |  |  |  |  |  |  |  |  |  |  |
| Ruminal pH | 32 | C | 6.804 | 0.085 | 0.001 | 0.008 | 0.868 | 0.00002 | 0.0003 | 0.949 | -1.16×10^-7^ | 1.89×10^-6^ | 0.951 | 0.54 | 0.17 |
| C_2_ (g/100 g VFA) | 21 | C | 65.86 | 4.314 | -0.09 | 0.205 | 0.666 | 0.006 | 0.006 | 0.301 | -0.0001 | 0.00004 | 0.184 | 0.60 | 4.53 |
| C_3_ (g/100 g VFA) | 21 | C | 21.08 | 0.742 | 0.071 | 0.114 | 0.542 | -0.004 | 0.004 | 0.312 | 0.00003 | 0.00001 | 0.292 | 0.43 | 1.31 |
| C_3_ (mmol/L) | 14 | C | 20.14 | 0.787 | 0.539 | 0.066 | <0.01 | -0.027 | 0.004 | <0.01 | 0.0003 | 0.0001 | <0.01 | 0.61 | 1.44 |
| IVOMD (%) | 34 | C | 64.05 | 6.325 | -0.258 | 0.777 | 0.742 | 0.009 | 0.025 | 0.730 | -0.0001 | 0.0002 | 0.629 | 0.52 | 12.0 |
| CH_4_/total gas | 25 | C | 0.098 | 0.028 | 0.001 | 0.001 | 0.631 | -0.00002 | 0.00004 | 0.667 | 9.14×10^-8^ | 0.000001 | 0.728 | 0.57 | 0.04 |

The model tended to be significantly compatible at *a P*-value≤0.1. The model is considered compatible at a *P*-value≤0.05. n, number of observations; SE, standard error; ^a^L, linear; Q, quadratic; C, cubic. ^b^Root mean square error; C_2_, acetate; C_3_, propionate; NH_3_, ammonia; IVOMD, in vitro organic matter digestibility; TGP, total gas production; CH_4_, methane.

**Table SM2 Regression equations established between a selected insect (*Gryllus bimaculatus*) intake dose (x, g) and measurable response (y) in vivo trials**

| **Response Parameters** | **Model^a^** | **n** | **Parameter estimates** | | | | | | | | | | |  | **Adjusted R^2^** | **RMSE^b^** |
| --- | --- | --- | --- | --- | --- | --- | --- | --- | --- | --- | --- | --- | --- | --- | --- | --- |
|  |  |  | **β0** | **SE** | **β1** | **SE** | **P value** | **β2** | **SE** | **P value** | **β3** | **SE** | **P value** |  |  |  |
| Feed efficiency | C | 10 | 14.8 | 2.80 | 0.647 | 0.314 | 0.175 | -0.018 | 0.007 | 0.125 | 0.000093 | 0.000033 | 0.108 |  | 0.74 | 4.06 |
| Ruminal pH | Q | 8 | 6.67 | 0.07 | 0.004 | 0.002 | 0.212 | -0.00002 | 0.00002 | 0.362 |  |  |  |  | 0.62 | 5.01 |
| Ammonia-nitrogen (mg %) | Q | 8 | 18.6 | 2.56 | 0.055 | 0.005 | 0.009 | -0.0002 | 0.00003 | 0.032 |  |  |  |  | 0.72 | 0.49 |
| Protozoa (×10^7^ /mL) | Q | 8 | 0.44 | 0.11 | -0.0005 | 0.001 | 0.744 | -0.000001 | 0.00001 | 0.874 |  |  |  |  | 0.75 | 1.56 |
| Total bacteria (×10^11^/mL) | C | 7 | 6.46 | 1.56 | 0.001 | 0.027 | 0.964 | 0.0002 | 0.00048 | 0.709 | 9.6×10^-7^ | 2×10^-6^ | 0.678 |  | 0.95 | 10.8 |
| TVFA (mmol/L) | C | 11 | 93.5 | 6.78 | 0.955 | 0.444 | 0.098 | -0.012 | 0.008 | 0.203 | 0.000045 | 0.000035 | 0.276 |  | 0.77 | 13.3 |
| C_2_ (g/100 g VFA) | C | 10 | 60.4 | 5.77 | -0.053 | 0.234 | 0.830 | 0.001 | 0.004 | 0.827 | 4.84×10^-6^ | 0.000019 | 0.807 |  | 0.79 | 7.69 |
| C_3_ (g/100 g VFA) | Q | 10 | 28.5 | 4.41 | 0.042 | 0.042 | 0.368 | -0.0002 | 0.0003 | 0.482 |  |  |  |  | 0.65 | 5.79 |
| C_4_ (g/100 g VFA) | C | 10 | 13.0 | 1.60 | -0.018 | 0.096 | 0.861 | -0.001 | 0.002 | 0.768 | 3.36×10^-6^ | 7.52×10^-6^ | 0.678 |  | 0.92 | 7.04 |
| C_2_ (mmol/L) | Q | 6 | 63.6 | 6.96 | 0.240 | 0.189 | 0.425 | -0.0003 | 0.0012 | 0.838 |  |  |  |  | 0.70 | 16.8 |
| C_3_ (mmol/L) | Q | 6 | 22.1 | 6.23 | 0.107 | 0.108 | 0.503 | -0.00003 | 0.0006 | 0.969 |  |  |  |  | 0.75 | 6.43 |
| C_4_ (mmol/L) | Q | 6 | 8.68 | 0.94 | 0.211 | 0.069 | 0.202 | -0.002 | 0.0008 | 0.249 |  |  |  |  | 0.53 | 2.79 |
| C_2_:C_3_ | Q | 6 | 3.42 | 0.13 | -0.010 | 0.002 | 0.030 | 0.00002 | 0.00001 | 0.139 |  |  |  |  | 0.86 | 29.5 |
| ADFD (%) | Q | 8 | 38.4 | 2.67 | 0.055 | 0.024 | 0.154 | -0.0002 | 0.0001 | 0.219 |  |  |  |  | 0.44 | 4.68 |

The model tended to be significantly compatible at a *P*-value≤0.1. The model is considered compatible at a *P*-value≤0.05. n, number of observations; SE, standard error; ^a^ Q, quadratic; C, cubic. ^b^Root mean square error; TVFA, total volatile fatty acids; C_2_, acetate; C_3_, propionate; C_4_, butyrate; ADFD, acid detergent fibre digestibility.

References

1. Yanza YR, Szumacher-Strabel M, Jayanegara A, Kasenta AM, Gao M, Huang H, Patra AK, Warzych E, Cieślak A. The effects of dietary medium-chain fatty acids on ruminal methanogenesis and fermentation in vitro and in vivo: A meta-analysis. *Journal of Animal Physiology and Animal Nutrition* (2021) 105:874–889. doi: 10.1111/jpn.13367

2. Jayanegara A, Wina E, Takahashi J. Meta-analysis on Methane Mitigating Properties of Saponin-rich Sources in the Rumen: Influence of Addition Levels and Plant Sources. *Asian-Australas J Anim Sci* (2014) 27:1426–1435. doi: 10.5713/ajas.2014.14086

3. Toral PG, Hervás G, González-Rosales MG, Mendoza AG, Robles-Jiménez LE, Frutos P. Insects as alternative feed for ruminants: comparison of protein evaluation methods. *J Animal Sci Biotechnol* (2022) 13:21. doi: 10.1186/s40104-021-00671-2

4. Schwarzer G. meta: An R package for meta-analysis. *R news* (2007) 7:40–45. https://cran.rstudio.org/doc/Rnews/Rnews_2007-3.pdf#page=40 [Accessed August 20, 2024]

5. Viechtbauer W. Conducting Meta-Analyses in R with the metafor Package. *Journal of Statistical Software* (2010) 36:1–48. doi: 10.18637/jss.v036.i03

6. Higgins JPT, Thompson SG. Quantifying heterogeneity in a meta-analysis. *Statistics in Medicine* (2002) 21:1539–1558. doi: 10.1002/sim.1186

7. Ningsih N, Respati AN, Astuti D, Triswanto T, Purnamayanti L, Yano AA, Putra RP, Jayanegara A, Ratriyanto A, Irawan A. Efficacy of *Bacillus subtilis* to replace in-feed antibiotics of broiler chickens under necrotic enteritis-challenged experiments: a systematic review and meta-analysis. *Poultry Science* (2023) 102:102923. doi: 10.1016/j.psj.2023.102923
