# Supplementary material for: Cutting-edge exploration of insect utilization in ruminant nutrition—feature and future: a systematic review and meta-analysis
Source: Front Vet Sci. 2024 Nov 20;11:1484870. doi: 10.3389/fvets.2024.1484870 (PMC11616318; doi:10.3389/fvets.2024.1484870)
Supplement: SUPPLEMENTARY MATERIAL 2 — Datasheet. [file Supplementary_file_2.docx]

**Table S1 In vitro experiments included in the review of the effect of various insects on ruminal fermentations**

| **No** | **Study^1^** | **n** | **Method** | **Incubation time** | **Animal donors** | **Animal status** |
| --- | --- | --- | --- | --- | --- | --- |
| 1 | (1) | 20 | BC | 24 h | Non-lactating Holstein cows | (8 years old, 894 kg of BW) |
| 2 | (3) | 6 | BC | 48 h | Friesian Holstein cow | NR |
| 3 | (18) | 6 | BC | 48 h | Non-lactating fistulated Friesian-Holstein cow | NR |
| 4 | (19) | 12 | BC | 24 h | Non-lactating Holstein cows | (7 years old, 894 kg of BW) |
| 5 | (21) | 8 | BC | 24 h | Cannulated Texel Sheep | (adult castrated males, 64.6 ± 7.10 kg of BW) |
| 6 | (23) | 9 | BC | 96 h | Rumen-fistulated dairy steers (75%  Holstein Friesian and 25% Thai native breed) | (3 years old, 320±10 kg of BW) |
| 7 | (24) | 30 | BC | 48 h | Ruminally cannulated non-lactating Holstein cows | (3 years old, 530 kg of BW) |
| 8 | (27) (exp.1) | 6 | BC | 48 h | Ettawa crossbred goats | NR |
| 9 | (27) (exp.2) | 6 | BC | 48 h | Ettawa crossbred goats | NR |
| 10 | (29) | 9 | GS | 24 h | Cattle with known history from the slaughterhouse | NR |
| 11 | (31) | 9 | BC | 96 h | Fistulated dairy bulls | 450 ± 30 kg of BW |
| 12 | (32) | 6 | BC | 48 h | Friesian Holstein cow | NR |
| 13 | (33) | 9 | GS | 96 h | NR | NR |
| 14 | (34) | 5 | BC | 48 h | Non-lactating Holstein Friesian cows | NR |

^1^ The references are cited throughout the main body of the manuscript; BC, batch culture - Serum bottles study; GS, glass syringes; NR, not report.

**Table S2 In vitro experiments included in the review of the effect of various insects on ruminal fermentations**

| **Inspects species** | **n** | **Buffer/bottle (mL)** | **Substrate (g)** | **Insects, g/bottle** | **Insects, %/bottle** | **Substrates form** | **Instar/stage** | **Reference^1^** |
| --- | --- | --- | --- | --- | --- | --- | --- | --- |
| *Blatta lateralis** | 8 | 40 | 0.6 | 0.6 | 100 | meal | subadult | (21) |
| *Brachytrupes portentosus* | 12 | 60 | 0.5 | 0.05 | 10 | meal | adult | (19) |
| *Grylloides sigillatus** | 8 | 40 | 0.6 | 0.6 | 100 | meal | adult | (21) |
| *Gryllus assimilis** | 6 | 75 | 0.75 | 0.75 | 100 | meal | 7 wk | (3) |
| *Musca domestica** | 8 | 40 | 0.6 | 0.6 | 100 | meal | larval | (21) |
| *Acheta domesticus** | 8 | 40 | 0.6 | 0.6 | 100 | meal | adult | (21) |
| *Acheta domesticus* | 12 | 60 | 0.5 | 0.05 | 10 | meal | adult | (19) |
| *Acheta domesticus** | 8 | 40 | 0.6 | 0.6 | 100 | meal | larval | (21) |
| *Tenebrio molitor** | 8 | 40 | 0.6 | 0.6 | 100 | meal | larval | (21) |
| *Tenebrio molitor* | 9 | 30 | 0.2 | 0.001 | 0.5 | meal | larvae | (29) |
| *Tenebrio molitor* | 9 | 30 | 0.2 | 0.002 | 1 | meal | larvae | (29) |
| *Tenebrio molitor* | 9 | 30 | 0.2 | 0.003 | 1.5 | meal | larvae | (29) |
| *Tenebrio molitor** | 6 | 75 | 0.75 | 0.75 | 100 | meal | 4 wk | (3) |
| *Bombyx mori* | 20 | 60 | 0.5 | 0.05 | 10 | meal | pupae | (1) |
| *Bombyx mori* | 20 | 60 | 0.5 | 0.1 | 20 | meal | pupae | (1) |
| *Bombyx mori* | 20 | 60 | 0.5 | 0.15 | 30 | meal | pupae | (1) |
| *Bombyx mori* | 20 | 60 | 0.5 | 0.2 | 40 | meal | pupae | (1) |
| *Bombyx mori* | 12 | 60 | 0.5 | 0.05 | 10 | meal | pupae | (19) |
| *Hermetia illucens** | 6 | 75 | 0.75 | 0.75 | 100 | meal | 1 wk | (3) |
| *Hermetia illucens** | 6 | 75 | 0.75 | 0.75 | 100 | meal | 2 wk | (3) |
| *Hermetia illucens* | 6 | 75 | 0.75 | 0.3 | 40 | meal | 1 wk | (18) |
| *Hermetia illucens* | 6 | 75 | 0.75 | 0.3 | 40 | meal | 2 wk | (18) |
| *Hermetia illucens* | 6 | 75 | 0.75 | 0.3 | 40 | meal | 1 wk | (18) |
| *Hermetia illucens* | 6 | 75 | 0.75 | 0.3 | 40 | meal | 2 wk | (18) |
| *Hermetia illucens* | 30 | 60 | 0.46 | 0.01472 | 3.2 | meal | larvae | (24) |
| *Hermetia illucens* | 30 | 60 | 0.46 | 0.02944 | 6.4 | meal | larvae | (24) |
| *Hermetia illucens** | 8 | 40 | 0.6 | 0.6 | 100 | meal | larval | (21) |
| *Gryllus bimaculatus** | 8 | 40 | 0.6 | 0.6 | 100 | meal | adult | (21) |
| *Gryllus bimaculatus* | 9 | 40 | 0.5 | 0.35 | 70 | meal | 30 - 35 d | (31) |
| *Gryllus bimaculatus* | 9 | 40 | 0.5 | 0.3465 | 69.3 | meal | 30 - 35 d | (31) |
| *Gryllus bimaculatus* | 9 | 40 | 0.5 | 0.343 | 68.6 | meal | 30 - 35 d | (31) |
| *Gryllus bimaculatus* | 9 | 40 | 0.5 | 0.3395 | 67.9 | meal | 30 - 35 d | (31) |
| *Gryllus bimaculatus* | 9 | 40 | 0.5 | 0.336 | 67.2 | meal | 30 - 35 d | (31) |
| *Gryllus bimaculatus* | 9 | 40 | 0.5 | 0.3325 | 66.5 | meal | 30 - 35 d | (31) |
| *Gryllus bimaculatus* | 9 | 40 | 0.5 | 0.329 | 65.8 | meal | 30 - 35 d | (31) |
| *Gryllus bimaculatus* | 9 | 40 | 0.5 | 0.3255 | 65.1 | meal | 30 - 35 d | (31) |
| *Gryllus bimaculatus* | 12 | 60 | 0.5 | 0.05 | 10 | meal | adult | (19) |
| *Gryllus bimaculatus* | 20 | 60 | 0.5 | 0.05 | 10 | meal | adult | (1) |
| *Gryllus bimaculatus* | 20 | 60 | 0.5 | 0.1 | 20 | meal | adult | (1) |
| *Gryllus bimaculatus* | 20 | 60 | 0.5 | 0.15 | 30 | meal | adult | (1) |
| *Gryllus bimaculatus* | 20 | 60 | 0.5 | 0.2 | 40 | meal | adult | (1) |
| *Gryllus bimaculatus* | 9 | 30 | 0.2 | 0.0035 | 1.76 | meal | adult | (23) |
| *Gryllus bimaculatus* | 9 | 30 | 0.2 | 0.0069 | 3.468 | meal | adult | (23) |
| *Gryllus bimaculatus* | 9 | 30 | 0.2 | 0.0104 | 5.176 | meal | adult | (23) |
| *Gryllus bimaculatus* | 9 | 30 | 0.2 | 0.0137 | 6.872 | meal | adult | (23) |
| *Gryllus bimaculatus* | 9 | 30 | 0.2 | 0.0053 | 2.64 | meal | adult | (23) |
| *Gryllus bimaculatus* | 9 | 30 | 0.2 | 0.0104 | 5.202 | meal | adult | (23) |
| *Gryllus bimaculatus* | 9 | 30 | 0.2 | 0.0155 | 7.764 | meal | adult | (23) |
| *Gryllus bimaculatus* | 9 | 30 | 0.2 | 0.0206 | 10.308 | meal | adult | (23) |
| ***Reference substrate*** |  |  |  |  |  |  |  |  |
| Soybean Meal (SBM)* | 6 | 75 | 0.75 | NA | 0 | meal | - | (3) |
| SBM* | 8 | 40 | 0.6 | NA | 0 | meal | - | (21) |
| Rapeseed meal (RPM)* | 8 | 40 | 0.6 | NA | 0 | meal | - | (21) |
| Sunflower meal (SFM)* | 8 | 40 | 0.6 | NA | 0 | meal | - | (21) |
| Fishmeal (FM)* | 8 | 40 | 0.6 | NA | 0 | meal | - | (21) |
| Napier grass* | 6 | 75 | 0.75 | NA | 0 | meal | - | (18) |
| Kleingrass hay* | 12 | 60 | 0.5 | NA | 0 | meal | - | (19) |
| Alfalfa hay* | 9 | 30 | 0.2 | NA | 0 | meal | - | (29) |

^1^ The references are cited throughout the main body of the manuscript; *As the sole substrate in the fermentation media; NA, not applied.

**Table S3 In vitro experiments included in the review of the effect of various insects on ruminal fermentations**

| **Study^1^** | **Feeding ration for donor animals** | **Treatments (Insects meal as pure substrate, unless otherwise stated)** |
| --- | --- | --- |
| (1) | Orchard grass (Dactylis glomerata) hay | Control group - 300 mg grass hay + 200 mg concentrate mixture |
|  | Orchard grass (Dactylis glomerata) hay | 300 mg grass hay + 150 mg concentrate + 50 mg insect (10% field crickets - *Gryllus bimaculatus*) |
|  | Orchard grass (Dactylis glomerata) hay | 300 mg grass hay + 100 mg concentrate + 100 mg insect (20% field crickets - *Gryllus bimaculatus*) |
|  | Orchard grass (Dactylis glomerata) hay | 300 mg grass hay + 50 mg concentrate + 150 mg insect (30% field crickets - *Gryllus bimaculatus*) |
|  | Orchard grass (Dactylis glomerata) hay | 300 mg grass hay + 0 mg concentrate + 200 mg insect (40% field crickets - *Gryllus bimaculatus*) |
|  | Orchard grass (Dactylis glomerata) hay | 300 mg grass hay + 150 mg concentrate + 50 mg insect (10% Silkworm - *Bombyx mori*) |
|  | Orchard grass (Dactylis glomerata) hay | 300 mg grass hay + 100 mg concentrate + 100 mg insect (20% Silkworm - *Bombyx mori*) |
|  | Orchard grass (Dactylis glomerata) hay | 300 mg grass hay + 50 mg concentrate + 150 mg insect (30% Silkworm - *Bombyx mori*) |
|  | Orchard grass (Dactylis glomerata) hay | 300 mg grass hay + 0 mg concentrate + 200 mg insect (40% Silkworm - *Bombyx mori*) |
|  |  |  |
| (3) | NR | Soybean Meal (SBM) |
|  | NR | Jamaican field cricket (JFC, *Gryllus assimilis*) |
|  | NR | Mealworm (MW, *Tenebrio molitor* |
|  | NR | Black soldier fly larvae (BSF1, *Hermetia illucens*) |
|  | NR | Black soldier fly larvae (BSF2, *Hermetia illucens*) |
|  |  |  |
| (18) | NR | 100% napier grass |
|  | NR | 60% napier grass + 40% SBM |
|  | NR | 60% napier grass + 40% BSF1; [Black soldier fly larvae (BSF1, *Hermetia illucens*)] |
|  | NR | 60% napier grass + 40% BSF2; [Black soldier fly larvae (BSF2, *Hermetia illucens*)] |
|  | NR | 60% napier grass + 20% SBM + 20% BSF1 |
|  | NR | 60% napier grass + 20% SBM + 20% BSF2. |
|  |  |  |
| (19) | 30 kg corn silage/day + 1 kg Japanese white birch (Betula platyphylla)/day | 500 mg of Kleingrass hay (100% KG) |
|  | 30 kg corn silage/day + 1 kg Japanese white birch (Betula platyphylla)/day | 300 mg KG + 200 mg soybean meal (SBM) (60% KG + 40% SBM) |
|  | 30 kg corn silage/day + 1 kg Japanese white birch (Betula platyphylla)/day | 300 mg KG + 150 mg SBM + 50 mg *A.domesticus* (A.d) (60% KG + 30% SBM + 10% A.d); house crickets (*A. domesticus*) |
|  | 30 kg corn silage/day + 1 kg Japanese white birch (Betula platyphylla)/day | 300 mg KG + 150 mg SBM + 50 mg *B.portentosus* (B.p) (60% KG + 30% SBM + 10% B.p); giant crickets (*B. portentosus*) |
|  | 30 kg corn silage/day + 1 kg Japanese white birch (Betula platyphylla)/day | 300 mg KG + 150 mg SBM + 50 mg *G. bimaculatus* (G.b) (60% KG + 30% SBM + 10% G.b); field crickets (*G. bimaculatus*) |
|  | 30 kg corn silage/day + 1 kg Japanese white birch (Betula platyphylla)/day | 300 mg KG + 150 mg SBM + 50 mg *B. mori* (B.m) (60% KG + 30% SBM + 10% B.m); silkworm (*B. mori*) |
|  |  |  |
| (21) | permanent grassland hay (840 g dry matter (DM)/head/d) and concentrate (360 g DM/head/d) | *Acheta domesticus L. –* ACD |
|  | permanent grassland hay (840 g dry matter (DM)/head/d) and concentrate (360 g DM/head/d) | *Alphitobius diaperinus Panzer –* ALD |
|  | permanent grassland hay (840 g dry matter (DM)/head/d) and concentrate (360 g DM/head/d) | *Blatta lateralis Walker –* BL |
|  | permanent grassland hay (840 g dry matter (DM)/head/d) and concentrate (360 g DM/head/d) | *Gryllus bimaculatus De Geer –* GB |
|  | permanent grassland hay (840 g dry matter (DM)/head/d) and concentrate (360 g DM/head/d) | *Grylloides sigillatus Walker –* GS |
|  | permanent grassland hay (840 g dry matter (DM)/head/d) and concentrate (360 g DM/head/d) | *Hermetia illucens L. –* HI |
|  | permanent grassland hay (840 g dry matter (DM)/head/d) and concentrate (360 g DM/head/d) | *Musca domestica L. –* MD |
|  | permanent grassland hay (840 g dry matter (DM)/head/d) and concentrate (360 g DM/head/d) | *Tenebrio molitor L. –* TM |
|  | permanent grassland hay (840 g dry matter (DM)/head/d) and concentrate (360 g DM/head/d) | SBM |
|  | permanent grassland hay (840 g dry matter (DM)/head/d) and concentrate (360 g DM/head/d) | Rapeseed meal (RPM) |
|  | permanent grassland hay (840 g dry matter (DM)/head/d) and concentrate (360 g DM/head/d) | Sunflower meal (SFM) |
|  | permanent grassland hay (840 g dry matter (DM)/head/d) and concentrate (360 g DM/head/d) | Fishmeal (FM) |
|  |  |  |
| (23) | Fed with rice straw ad libitum and concentrate mixture (14% CP, 75% TDN) at 0.5% of body weight | Roughage to concentrate (R:C) ratio 60:40, replaced SBM with cricket meal (*Gryllus bimaculatus*) (CM) in a concentrate ratio at 0% |
|  | Fed with rice straw ad libitum and concentrate mixture (14% CP, 75% TDN) at 0.5% of body weight | Roughage to concentrate (R:C) ratio 60:40, replaced SBM with cricket meal (*Gryllus bimaculatus*) (CM) in a concentrate ratio at 25% |
|  | Fed with rice straw ad libitum and concentrate mixture (14% CP, 75% TDN) at 0.5% of body weight | Roughage to concentrate (R:C) ratio 60:40, replaced SBM with cricket meal (*Gryllus bimaculatus*) (CM) in a concentrate ratio at 50% |
|  | Fed with rice straw ad libitum and concentrate mixture (14% CP, 75% TDN) at 0.5% of body weight | Roughage to concentrate (R:C) ratio 60:40, replaced SBM with cricket meal (*Gryllus bimaculatus*) (CM) in a concentrate ratio at 75% |
|  | Fed with rice straw ad libitum and concentrate mixture (14% CP, 75% TDN) at 0.5% of body weight | Roughage to concentrate (R:C) ratio 60:40, replaced SBM with cricket meal (*Gryllus bimaculatus*) (CM) in a concentrate ratio at 100% |
|  | Fed with rice straw ad libitum and concentrate mixture (14% CP, 75% TDN) at 0.5% of body weight | Roughage to concentrate (R:C) ratio 40:60, replaced SBM with cricket meal (*Gryllus bimaculatus*) (CM) in a concentrate ratio at 0% |
|  | Fed with rice straw ad libitum and concentrate mixture (14% CP, 75% TDN) at 0.5% of body weight | Roughage to concentrate (R:C) ratio 40:60, replaced SBM with cricket meal (*Gryllus bimaculatus*) (CM) in a concentrate ratio at 25% |
|  | Fed with rice straw ad libitum and concentrate mixture (14% CP, 75% TDN) at 0.5% of body weight | Roughage to concentrate (R:C) ratio 40:60, replaced SBM with cricket meal (*Gryllus bimaculatus*) (CM) in a concentrate ratio at 50% |
|  | Fed with rice straw ad libitum and concentrate mixture (14% CP, 75% TDN) at 0.5% of body weight | Roughage to concentrate (R:C) ratio 40:60, replaced SBM with cricket meal (*Gryllus bimaculatus*) (CM) in a concentrate ratio at 75% |
|  | Fed with rice straw ad libitum and concentrate mixture (14% CP, 75% TDN) at 0.5% of body weight | Roughage to concentrate (R:C) ratio 40:60, replaced SBM with cricket meal (*Gryllus bimaculatus*) (CM) in a concentrate ratio at 100% |
|  |  |  |
| (24) | The cows were fed TMRs containing 13% CP, 93% OM, 45% NDF, 28% ADF, and 6% ADL | Roughage to concentrate (R:C) ratio 60:40, replaced SBM with black soldier fly (*Hermetia illucens*) in a TMR ratio at 0% |
|  | The cows were fed TMRs containing 13% CP, 93% OM, 45% NDF, 28% ADF, and 6% ADL | Roughage to concentrate (R:C) ratio 60:40, replaced SBM with black soldier fly (*Hermetia illucens*) in a TMR ratio at 20% |
|  | The cows were fed TMRs containing 13% CP, 93% OM, 45% NDF, 28% ADF, and 6% ADL | Roughage to concentrate (R:C) ratio 60:40, replaced SBM with black soldier fly (*Hermetia illucens*) in a TMR ratio at 40% |
|  |  |  |
| (27) (exp.1) | NR | Whole cricket C - as a sole substrate |
|  | NR | Cricket after exoskeleton removal (CER) - as a sole substrate |
|  | NR | Cricket after chemical extraction (CCE) - as a sole substrate |
|  |  |  |
| (27) (exp.2) | NR | Control meal - R1 |
|  | NR | Control meal + 15% Cricket meal - R2 |
|  | NR | Control meal + 30% Cricket meal - R3 |
|  | NR | Control meal + 15% cricket meal after exoskeleton removal - R4 |
|  |  |  |
| (29) | NR | C: %100 alfalfa hay |
|  | NR | MWL0.5: alfalfa + 0.5% yellow mealworm (*Tenebrio Molitor L.*) |
|  | NR | MWL1: alfalfa + 1% yellow mealworm (*Tenebrio Molitor L.*) |
|  | NR | MWL1.5: alfalfa + 1.5% yellow mealworm (*Tenebrio Molitor L.*) |
|  |  |  |
| (31) | TMR containing 14% and 75% of CP and TDN, respectively | T1 - Ratio of *G.bimaculatus to S.grandiflora* (100:0) 0.35g + 0.15g of rice straw |
|  | TMR containing 14% and 75% of CP and TDN, respectively | T2 - Ratio of *G.bimaculatus to S.grandiflora* (99:1) 0.35g + 0.15g of rice straw |
|  | TMR containing 14% and 75% of CP and TDN, respectively | T3 - Ratio of *G.bimaculatus to S.grandiflora* (98:2) 0.35g + 0.15g of rice straw |
|  | TMR containing 14% and 75% of CP and TDN, respectively | T4 - Ratio of *G.bimaculatus to S.grandiflora* (97:3) 0.35g + 0.15g of rice straw |
|  | TMR containing 14% and 75% of CP and TDN, respectively | T5 - Ratio of *G.bimaculatus to S.grandiflora* (96:4) 0.35g + 0.15g of rice straw |
|  | TMR containing 14% and 75% of CP and TDN, respectively | T6 - Ratio of *G.bimaculatus to S.grandiflora* (95:5) 0.35g + 0.15g of rice straw |
|  | TMR containing 14% and 75% of CP and TDN, respectively | T7 - Ratio of *G.bimaculatus to S.grandiflora* (94:6) 0.35g + 0.15g of rice straw |
|  | TMR containing 14% and 75% of CP and TDN, respectively | T8 - Ratio of *G.bimaculatus to S.grandiflora* (93:7) 0.35g + 0.15g of rice straw |
|  |  |  |
|  |  |  |
| (32) | NR | Grasshopper meal (GHM, *Melanoplus sanguinipes*) |
|  | NR | Earthworm meal (EWM, *Lumbricus rubellus*) |
|  | NR | Fish by-product meal (FbPM, *Canthidermis maculate*) |
|  | NR | Centipede meal (CM, *Scolopendra cataracta*) |
|  | NR | Snail meal (SM, *Pomacea canaliculata*) |
|  | NR | Ant eggs meal (AEM, *Solenopsis invicta*) |
|  | NR | Mealworm meal (MWM, *Tenebrio molitor*) |
|  | NR | SBM |
|  |  |  |
| (33) | NR | Black soldier fly (*Hermetia illucens*, BSF) |
|  | NR | Yellow mealworm (*Tenebrio molitor*, YMW) |
|  | NR | Aquatic insect Noctonecta spp. |
|  | NR | Fishmeal |
|  | NR | SBM |
|  |  |  |
| (34) | NR | Ensiled TMR (CON) |
|  | NR | Ensiled 80% TMR + 20% intact BSFL (black soldier fly larvae (BSFL)) (T2) |
|  | NR | Ensiled 80% TMR + 20% chemically defatted BSFL (T3) |
|  | NR | Ensiled 80% TMR + 20% mechanically defatted BSFL (T4) |

^1^ The references are cited throughout the main body of the manuscript; NR, not report.

**Table S4 In vivo experiments included in the review of the effect of various insects on ruminal fermentations**

| **No** | **Study^1^** | **n** | **Animal model** | **Experimental period** | **Treatments** |
| --- | --- | --- | --- | --- | --- |
| 1 | (4) | 4 | Four Thai native male beef cattle | 84 d | Control diet with SBM |
| 1 | (4) | 4 | Four Thai native male beef cattle | 84 d | Control diet with cricket (*Gryllus bimaculatus*) meal pellets replace 33% of SBM |
| 1 | (4) | 4 | Four Thai native male beef cattle | 84 d | Control diet with cricket (*Gryllus bimaculatus*) meal pellets replace 67% of SBM |
| 1 | (4) | 4 | Four Thai native male beef cattle | 84 d | Control diet with cricket (*Gryllus bimaculatus*) meal pellets replace 100% of SBM |
|  |  |  |  |  |  |
| 2 | (36) (exp.1) | 4 | Twelve pre-weaning Etawah crossbred goat kids | 56 d | Control - kids were given goat milk (GM) |
| 2 | (36) (exp.1) | 4 | Twelve pre-weaning Etawah crossbred goat kids | 56 d | Kids were given cow milk (COWM) |
| 2 | (36) (exp.1) | 4 | Twelve pre-weaning Etawah crossbred goat kids | 56 d | Kids were given milk replacer containing cricket meal (*Gryllus bimaculatus*) (MR) |
|  |  |  |  |  |  |
| 3 | (36) (exp.2) | 4 | Twelve post-weaning Etawah crossbred goats | 60 d | CM-0= concentrate without cricket meal but containing 30% soybean meal |
| 3 | (36) (exp.2) | 4 | Twelve post-weaning Etawah crossbred goats | 60 d | CM-15= concentrate containing 15% cricket meal and 15% soybean meal |
| 3 | (36) (exp.2) | 4 | Twelve post-weaning Etawah crossbred goats | 60 d | CM-30= concentrate containing 30% cricket meal without soybean meal |
|  |  |  |  |  |  |
| 4 | (37) | 4 | Twelve post weaning local lambs | 60 d | Control: fed 40% of forage *Brachiaria humidicola* and 60% of concentrate containing 15% soybean meal without cricket meal |
| 4 | (37) | 4 | Twelve post weaning local lambs | 60 d | T1: fed 40% of forage Brachiaria humidicola and 60% of concentrate containing 7.5% cricket meal in place of soybean meal |
| 4 | (37) | 4 | Twelve post weaning local lambs | 60 d | T2: fed 40% of forage Brachiaria humidicola and 60% of concentrate containing 15% cricket meal in place of soybean meal |
|  |  |  |  |  |  |
| 5 | (38) (exp.1) | 4 | Four crossbred steers | 60 d | T0 - The concentrate feed mixture containing SBM alone |
| 5 | (38) (exp.1) | 4 | Four crossbred steers | 60 d | T1 - The SBM was replaced by defatted silkworm pupae meal at levels of 10% |
| 5 | (38) (exp.1) | 4 | Four crossbred steers | 60 d | T2 - The SBM was replaced by defatted silkworm pupae meal at levels of 20% |
| 5 | (38) (exp.1) | 4 | Four crossbred steers | 60 d | T3 - The SBM was replaced by defatted silkworm pupae meal at levels of 30% |
|  |  |  |  |  |  |
| 6 | (38) (exp.2) | 5 | 20 male apparently healthy cattle | 30 d | T0 - The concentrate feed mixture containing SBM alone |
| 6 | (38) (exp.2) | 5 | 20 male apparently healthy cattle | 30 d | T1 - The SBM was replaced by defatted silkworm pupae meal at levels of 10% |
| 6 | (38) (exp.2) | 5 | 20 male apparently healthy cattle | 30 d | T2 - The SBM was replaced by defatted silkworm pupae meal at levels of 20% |
| 6 | (38) (exp.2) | 5 | 20 male apparently healthy cattle | 30 d | T3 - The SBM was replaced by defatted silkworm pupae meal at levels of 30% |
|  |  |  |  |  |  |
| 7 | (41) | 10 | Forty male Ossimi lambs | 120 d | T1 - Control ration (Con) consisted of 60% concentrate feed mixture (CFM) and + 40% rice straw (RS) |
| 7 | (41) | 10 | Forty male Ossimi lambs | 120 d | T2 - 10% Oriental Hornet Meal (OHM) |
| 7 | (41) | 10 | Forty male Ossimi lambs | 120 d | T3 - 20% Oriental Hornet Meal (OHM) |
| 7 | (41) | 10 | Forty male Ossimi lambs | 120 d | T4 - 40% Oriental Hornet Meal (OHM) |
|  |  |  |  |  |  |
| 8 | (43) | 4 | Twenty male sheep | 84 d | R1 (complete feed, control without BSF and bioconverted CPH) (BSF: black soldier fly; CPH: cocoa pod husk) |
| 8 | (43) | 4 | Twenty male sheep | 84 d | R2 (5% BSF) |
| 8 | (43) | 4 | Twenty male sheep | 84 d | R3 (20% bioconverted CPH) |
| 8 | (43) | 4 | Twenty male sheep | 84 d | R4 (2.5% BSF + 10% bioconverted CPH) |
| 8 | (43) | 4 | Twenty male sheep | 84 d | R5 (5% BSF + 20% bioconverted CPH) |

^1^ The references are cited throughout the main body of the manuscript

**Table S5 In vivo experiments included in the review of the effect of various insects on ruminal fermentations**

| **No** | **Study^1^** | **Ethical approval** | **Animal status** |
| --- | --- | --- | --- |
| 1 | (4) | Animal Care and Use Committee, Rajamangala University of Technology Isan, Thailand (approval no. 4/2565) | (Two years old, 230 ± 15 kg of BW) |
|  |  |  |  |
|  |  |  |  |
| 2 | (36) (exp.1) | Bogor Agricultural University Animal Welfare Committee with ACCUC No. 76-2017-IPB | (one week old, 3.78 ± 0.50 kg of BW) |
|  |  |  |  |
|  |  |  |  |
| 3 | (36) (exp.1) (exp.2) | Bogor Agricultural University Animal Welfare Committee with ACCUC No. 76-2017-IPB | (2 months old, 12 ± 0.40 kg of BW) |
|  |  |  |  |
|  |  |  |  |
| 4 | (37) | No specific statement | (2 months, 11.24 ± 1.62 kg of BW) |
|  |  |  |  |
| 5 | (38) (exp.1) | Approved by the Institutional Animal Ethics Committee constituted under CPCSEA, New Delhi, MoEFCC, Government of India. | (496.25 ± 5.39 kg of BW) |
|  |  |  |  |
|  |  |  |  |
| 6 | (38) (exp.2) | Approved by the Institutional Animal Ethics Committee constituted under CPCSEA, New Delhi, MoEFCC, Government of India. | (311.2 ± 4.81 kg of BW) |
|  |  |  |  |
|  |  |  |  |
| 7 | (41) | All studies were approved by Animal and Poultry Production Department, Faculty of Agriculture, Minia University. Ethics Number: MU/FA/014/12/22. | (20.58 ± 0.85 kg of BW) |
|  |  |  |  |
|  |  |  |  |
| 8 | (43) | No specific statement | (6–8 months, 20.42 ± 3.57 kg of BW) |

^1^ The references are cited throughout the main body of the manuscript

**Table S6 Descriptive statistics of the effects of *Gryllus bimaculatus, Bombyx mori, Acheta domesticus,* and *Hermetia illucens* on ruminal fermentation parameters in ruminants (*in vitro*)**

| **Items** | **Insects** | | | |
| --- | --- | --- | --- | --- |
|  | ***Gryllus bimaculatus*** | ***Bombyx mori*** | ***Acheta domesticus*** | ***Hermetia illucens*** |
| pH | 6.78±0.12 | 6.7±0.05 | 6.87±0.22 | 7.04±0.27 |
| NH_3_ (mg/dL) | 16.7±2.79 | 16.5±3.11 | 23.9 | 13.6±7.45 |
| TVFA (mmol/l) | 101±8.74 | 101±9.78 | 119 | 111±27.2 |
| C_2_ (g/100 g VFA) | 66.9±5.57 | 69.6±1.73 | 61.7±8.56 | 56.9 |
| C_3_ (g/100 g VFA) | 20.8±0.76 | 20.9±0.95 | 18.4±2.11 | 19.1 |
| C_4_ (g/100 g VFA) | 9.84±1.42 | 9.55±1.26 | 8.81±0.94 | 12.1 |
| C_2_ (mmol/l) | 69.7±7.27 | 70.5±8.01 | 85.3 | 45.8±0.46 |
| C_3_ (mmol/l) | 20.9±2.03 | 21.0±2.45 | 24.5 | 22.4±0.73 |
| C_4_ (mmol/l) | 9.92±1.0 | 9.56±0.71 | 9.16 | 0.45±0.02 |
| C_2_:C_3_ | 3.25±0.28 | 3.36±0.21 | 3.37±0.13 | 2.38±0.52 |
| IVDMD (%) | 52±11.3 | 37.0±5.64 | 45.6 | 56.4±6.48 |
| IVOMD (%) | 64.8±16.5 | 45.8 | 36.4±9.13 | 53.5±14.3 |
| IVNDFD (%) | 41.1 | 38.5 | 42.9 | 36.6±2.84 |
| IVADFD (%) | 27.3 | 35.2 | 34.8 | - |
| TGP (ml/g DM) | 95.2±14.2 | 92.4±9.45 | 88.8 | 147±32.7 |
| TGP (ml/g DDM) | 247±40.5 | 243±38.3 | 195 | - |
| CH_4_ (ml/g DM) | 6.18±1.29 | 5.81±0.78 | 5.39 | 39.7±14.4 |
| CH_4_ (ml/g DDM) | 16.1±3.63 | 15.5±3.18 | 11.8 | - |
| CO_2_ (ml/g DM) | 89±12.9 | 86.7±8.74 | 83.4 | - |
| CO_2_ (ml/g DDM) | 231±37.2 | 227±35.3 | 183 | - |
| CH_4_/TGP | 0.08±0.04 | 0.062±0.003 | 0.14±0.075 | - |

Abbreviation: SD, standard deviation; Max, maximum; Min, minimum; NH_3_, ammonia; TVFA, total volatile fatty acids; C_2_, acetate; C_3_, propionate; C_4_, butyrate; IVDMD, in vitro dry matter digestibility; IVOMD, in vitro organic matter digestibility; IVNDFD, in vitro neutral detergent fibre digestibility; IVADFD, in vitro acid detergent fibre digestibility; TGP, total gas production; DM, dry matter; DDM, degraded dry matter; CH_4_, methane; CO_2_, carbon dioxide. All values computed here are sourced from the literature listed in additional file 1.

**Table S7 Responses of ruminal fermentation parameters affected by different insects in the in vitro experiments**

| **Response parameters** | **n** | **Control** | ***Acheta domesticus*** | ***Tenebrio molitor*** | ***Bombyx mori*** | ***Hermetia illucens*** | ***Gryllus bimaculatus*** | **SEM** | ***P*-value** |
| --- | --- | --- | --- | --- | --- | --- | --- | --- | --- |
| pH | 37 | 6.73^ab^ | 6.87^ab^ | 7.13^a^ | 6.70^b^ | 7.04^ab^ | 6.78^ab^ | 0.04 | 0.02 |
| TVFA (mmol/l) | 28 | 97.1 | 119 | 128 | 101.2 | 110.6 | 100.6 | 3.94 | 0.62 |
| C_2_ (g/100 g VFA) | 23 | 63.1 | 61.7 | 55.4 | 69.6 | 56.9 | 66.9 | 1.40 | 0.17 |
| C_3_ (g/100 g VFA) | 23 | 21.3 | 18.4 | 20.4 | 20.9 | 19.1 | 20.8 | 0.34 | 0.12 |
| C_4_ (g/100 g VFA) | 23 | 8.95 | 8.81 | 9.10 | 9.55 | 12.1 | 9.84 | 0.30 | 0.41 |
| C_2_ (mmol/l) | 18 | 69.9^a^ | - | - | 70.5^a^ | 45.8^b^ | 69.7^a^ | 2.72 | 0.03 |
| C_3_ (mmol/l) | 19 | 22.4 | 24.5 | - | 21.0 | 22.4 | 20.9 | 0.49 | 0.45 |
| C_4_ (mmol/l) | 19 | 7.97 | 9.16 | - | 9.56 | - | 9.92 | 0.58 | 0.59 |
| C_2_:C_3_ | 26 | 2.97^a^ | 3.37^a^ | - | 3.36^a^ | 2.38^b^ | 3.25^a^ | 0.09 | 0.03 |
| NH_3_ (mg/dL) | 28 | 14.9 | 23.9 | 30.3 | 16.5 | 13.6 | 16.7 | 1.34 | 0.26 |
| IVDMD (%) | 44 | 56.2 | 45.6 | 59.6 | 37.0 | 56.4 | 52.0 | 1.87 | 0.07 |
| IVOMD (%) | 35 | 56.3 | 36.4 | 57.3 | 45.8 | 53.5 | 64.8 | 2.79 | 0.17 |
| TGP (ml/g DM) | 28 | 139^bc^ | - | 198^a^ | 92.4^c^ | 147.4^b^ | 95.2^c^ | 9.57 | <0.01 |
| TGP (ml/g DDM) | 14 | 215.4 | 194.8 | - | 242.8 | - | 247.2 | 11.1 | 0.58 |
| CH_4_ (ml/g DDM) | 13 | 12.5 | - | - | 15.5 | - | 16.1 | 1.02 | 0.43 |
| CO_2_ (ml/g DM) | 14 | 83.6 | 83.4 | - | 86.7 | - | 89.0 | 3.18 | 0.94 |
| CO_2_ (ml/g DDM) | 14 | 202.7 | 183 | - | 227.4 | - | 231.2 | 10.1 | 0.58 |
| CH_4_/TGP | 27 | 0.13 | 0.14 | 0.12 | 0.06 | 0.17 | 0.08 | 0.01 | 0.13 |

Different superscript alphabets of means in a row are significant differences at *P*-value≤0.05. SEM, standard error of the mean; NH_3_, ammonia; TVFA, total volatile fatty acids; C_2_, acetate; C_3_, propionate; C_4_, butyrate; IVDMD, in vitro dry matter digestibility; IVOMD, in vitro organic matter digestibility; TGP, total gas production; DM, dry matter; DDM, degraded dry matter; CH_4_, methane; CO_2_, carbon dioxide.

**Table S8 Descriptive statistics of the variables in the database used to evaluate the effect of various insects on blood biochemical parameters of ruminants (*in vivo*)**

| **Item** | **Unit** | **Insects** | | | |
| --- | --- | --- | --- | --- | --- |
|  |  | ***Hermetia illucens*** | ***Tenebrio molitor*** | ***Bombyx mori*** | ***Vespa Orientalis*** |
| ALP | (IU/L) | 83.3±13.8 | 281±17.2 | 110±15.4 | - |
| AST | (IU/L) | 63.9±3.24 | 38.2±8.25 | 83.8±0.64 | 39±0.27 |
| Albumin | (g/dL) | 3.36±0.1 | - | 3.23±1.21 | 4.39±0.23 |
| Globulin | (g/dL) | 5.69±0.07 | - | 2.44±0.22 | 2.4±0.14 |
| Total protein | (g/dL) | 9.05±0.03 | 7.33±0.42 | 5.82±1.18 | 6.8±0.37 |
| Phosphorus | (mM/L) | 2.08±0.08 | 1.81±0.17 | 2.86±0.18 | - |
| Magnesium | (mM/L) | 0.52±0.04 |  | 1.07±0.01 | - |
| Calcium | (mM/L) | 2.78±0.06 | 3.17±0.61 | 2.31±0.08 | - |
| BUN | (mg/dL) | 24.3±5.47 | 27.4±1.29 | 20.2±8.25 | 43.8±0.38 |
| Glucose | (mg/dL) | 47±10.5 | 64.2±6.36 | 38.2±1.07 | 75.1±1.78 |
| Cholesterol | (mg/dL) | 229±64.8 | - | 114±8.21 | 173±4.06 |
| Creatinine | (mg/dL) | - | - | 1.97±0.13 | 0.89±0.06 |
| ALT | (IU/L) | 24.6±0.76 | 73±32.5 | - | - |
| HCT | (%) | 36.5±0.83 | 30.8±2.75 | - | - |
| RBC | (10^12^/L) | 12.4±4.38 | 9.59±0.89 | - | - |
| WBC | (10^9^/L) | 11.2±2.4 | 7.54±0.66 | - | - |
| HGB | (g/L) | 91.9±2.55 | 123±4.7 | - | - |
| Triglyceride | (mg/dL) | 28.8±0.63 | 52.2±11.1 | - | - |

ALP, alkaline phosphatase; AST, aspartate transaminase; BUN, blood urea nitrogen; ALT, alanine transaminase; HCT: hematocrit test; RBC, red blood cell; WBC, white blood cells; HGB, Hemoglobin. The mean values for *Hermetia illucens* and *Tenebrio molitor*, derived from 24 and 6 experimental units, respectively, were computed based on the original studies’ findings. All values computed here are sourced from the literature in additional file 1.

**Table S9 Descriptive statistics of the variables in the database used to evaluate the effect of *Gryllus bimaculatus, Hermetia illucens,* and *Bombyx mori* on ruminal fermentation parameters in ruminants (*in vivo*)**

| **Item** | ***Gryllus bimaculatus*** | | | |  | ***Hermetia illucens*** | | | |  | ***Bombyx mori*** | | | |
| --- | --- | --- | --- | --- | --- | --- | --- | --- | --- | --- | --- | --- | --- | --- |
|  | **Mean** | **SD** | **Max** | **Min** |  | **Mean** | **SD** | **Max** | **Min** |  | **Mean** | **SD** | **Max** | **Min** |
| Growth performance |  |  |  |  |  |  |  |  |  |  |  |  |  |  |
| Feed efficiency | 15.3 | 8.80 | 23.2 | 0.54 |  | 11.5 | 1.2 | 12.8 | 10.6 |  | - | - | - | - |
| Ruminal fermentation |  |  |  |  |  |  |  |  |  |  |  |  |  |  |
| Ruminal pH | 6.91 | 0.09 | 6.98 | 6.81 |  | 6.62 | 0.24 | 6.85 | 6.39 |  | 6.62 | 0.02 | 6.64 | 6.60 |
| Ammonia-nitrogen (mg %) | 17.2 | 1.15 | 18.4 | 16.1 |  | 11.7 | 0.06 | 11.74 | 11.66 |  | 19.2 | 0.15 | 19.3 | 19.0 |
| NH_3_ (mg/dL) | 12.3 | 1.25 | 13.2 | 11.5 |  | - | - | - | - |  | 18.8 | 0.28 | 19.0 | 18.6 |
| Protozoa (×10^7^/mL) | 0.48 | 0.06 | 0.53 | 0.41 |  | - | - | - | - |  | 7.87 | 0.21 | 8.01 | 7.72 |
| Total bacteria (×10^11^/mL) | 9.10 | 0.55 | 9.49 | 8.71 |  | 5.76 | 1.25 | 6.64 | 4.87 |  | - | - | - | - |
| TVFA (mmol/L) | 117 | 15.8 | 136 | 97.1 |  | 101.2 | 16.4 | 123.3 | 86.6 |  | 72.2 | 9.04 | 83.5 | 62.8 |
| C_2_ (g/100 g VFA) | 57.3 | 7.62 | 64.5 | 48.3 |  | - | - | - | - |  | - | - | - | - |
| C_3_ (g/100 g VFA) | 32.5 | 7.39 | 41.5 | 26.0 |  | - | - | - | - |  | - | - | - | - |
| C_4_ (g/100 g VFA) | 10.2 | 1.19 | 11.3 | 8.51 |  | - | - | - | - |  | - | - | - | - |
| C_2_ (mmol/L) | 80.2 | 10.2 | 87.5 | 73.0 |  | - | - | - | - |  | 51.4 | 6.48 | 58.9 | 44.4 |
| C_3_ (mmol/L) | 33.4 | 4.41 | 36.6 | 30.3 |  | - | - | - | - |  | 12.1 | 0.88 | 13.3 | 11.2 |
| C_4_ (mmol/L) | 12.3 | 1.05 | 13.0 | 11.5 |  | - | - | - | - |  | 6.44 | 1.45 | 8.68 | 5.37 |
| C_2_:C_3_ | 2.57 | 0.31 | 2.90 | 2.30 |  | - | - | - | - |  | 3.93 | 0.05 | 3.96 | 3.89 |
| Apparent digestibility (%) |  |  |  |  |  |  |  |  |  |  |  |  |  |  |
| DM | 65.1 | 2.49 | 67.8 | 62.3 |  | - | - | - | - |  | 63.6 | 3.77 | 68.5 | 60.6 |
| OM | 64.3 | 3.11 | 67.5 | 61.3 |  | - | - | - | - |  | 67.0 | 1.67 | 69.4 | 65.3 |
| CP | 70.2 | 3.95 | 74.5 | 65.3 |  | - | - | - | - |  | 65.9 | 2.5 | 68.7 | 63.9 |
| NDF | 48.0 | 1.70 | 49.3 | 46.1 |  | - | - | - | - |  | 59.3 | 2.17 | 62.6 | 57.0 |
| ADF | 33.9 | 1.88 | 35.1 | 31.7 |  | - | - | - | - |  | 44.7 | 3.02 | 48.6 | 42.2 |

SD, standard deviation; Max, maximum; Min, minimum; NH_3_, ammonia; TVFA, total volatile fatty acids; C_2_, acetate; C_3_, propionate; C_4_, butyrate; DM, dry matter; OM, organic matter; CP, crude protein; NDF, neutral detergent fibre; ADF, acid detergent fibre. The mean values for *Gryllus bimaculatus, Hermetia illucens, and Bombyx mori*, derived from 64, 12, and 48 observations, respectively, were computed based on the original studies' findings.

**Table S10 Responses of ruminal fermentation parameters affected by different insects in the in vivo experiments**

| **Item** | **n** | **Control** | ***Gryllus bimaculatus*** | ***Bombyx mori*** | ***Vespa Orientalis*** | **SEM** | ***P*-value** |
| --- | --- | --- | --- | --- | --- | --- | --- |
| **Growth performance** |  |  |  |  |  |  |  |
| Feed efficiency | 16 | 13.9 | 15.3 | - | 6.49 | 1.92 | 0.26 |
| Ruminal fermentation |  |  |  |  |  |  |  |
| Ruminal pH | 14 | 6.65^b^ | 6.90^a^ | 6.62^b^ | 6.51^b^ | 0.05 | <0.01 |
| Ammonia-nitrogen (mg %) | 14 | 20.6 | 17.0 | 19.2 | 21.1 | 0.97 | 0.41 |
| Protozoa (×10^7^ cell/mL) | 11 | 0.44^ab^ | 0.50^a^ | - | 0.16^b^ | 0.06 | 0.03 |
| Total bacteria (cfu/mL) | 7 | 6.47 | 7.28 | - | - | 0.69 | 0.64 |
| TVFA (mmol/L) | 14 | 94.3^ab^ | 112^a^ | 78.4^b^ | - | 4.76 | 0.006 |
| C_2_ (g/100 g VFA) | 10 | 60.2 | 60.3 | - | - | 2.65 | 0.98 |
| C_3_ (g/100 g VFA) | 10 | 28.8 | 29.3 | - | - | 2.04 | 0.91 |
| C_4_ (g/100 g VFA) | 10 | 12.9 | 10.5 | - | - | 0.79 | 0.17 |
| C_2_ (mmol/L) | 9 | 66.2^ab^ | 80.2^a^ | 56.0^b^ | - | 4.27 | 0.01 |
| C_3_ (mmol/L) | 9 | 22.6^ab^ | 33.4^a^ | 12.6^b^ | - | 3.63 | 0.009 |
| C_4_ (mmol/L) | 9 | 9.38^ab^ | 12.3^a^ | 7.07^b^ | - | 0.88 | 0.004 |
| Apparent digestibility (%) |  |  |  |  |  |  |  |
| DM | 16 | 65.3^b^ | 65.1^b^ | 60.9^b^ | 72.3^a^ | 1.12 | 0.003 |
| OM | 14 | 63.4 | 64.3 | 65.9 | 63.9 | 0.56 | 0.55 |
| CP | 17 | 68.6^ab^ | 69.4^ab^ | 64.1^b^ | 76.3^a^ | 1.29 | 0.02 |
| EE | 8 | 65.9 | - | 67.8 | 64.3 | 0.77 | 0.14 |
| NDF | 14 | 52.4^b^ | 48.4^b^ | 58.0^a^ | 50.9^b^ | 1.07 | <0.01 |
| ADF | 14 | 40.3^ab^ | 35.0^b^ | 42.6^ab^ | 46.6^a^ | 1.47 | 0.007 |

Different superscript alphabets of means in a row are significant differences at *P*-value≤0.05. SEM, standard error of the mean; TVFA, total volatile fatty acids; C_2_, acetate; C_3_, propionate; C_4_, butyrate; DM, dry matter; OM, organic matter; CP, crude protein; EE, either extract; NDF, neutral detergent fibre; ADF, acid detergent fibre.

**Table S11 Responses of biochemical parameters of the blood affected by different insects in the in vivo experiments**

| **Item** | **Unit** | **n** | **Control** | ***Bombyx mori*** | ***Vespa Orientalis*** | ***Gryllus bimaculatus*** | **SEM** | ***P*-value** |
| --- | --- | --- | --- | --- | --- | --- | --- | --- |
| ALT | (U/l) | 5 | 67.5 | - | 26.4 | - | 16.3 | 0.27 |
| Albumin | (g/dL) | 8 | 3.13^ab^ | 2.35^b^ | 4.39^a^ | - | 0.38 | 0.02 |
| Globulin | (g/dL) | 8 | 2.54 | 2.38 | 2.40 | - | 0.09 | 0.83 |
| Total protein | (g/dL) | 12 | 6.15^ab^ | 4.97^b^ | 6.80^a^ | 6.71^a^ | 0.26 | 0.02 |
| BUN | (mg/dL) | 9 | 24.1^ab^ | 14.3^b^ | 43.8^a^ | - | 5.08 | 0.02 |
| Triglyceride | (mg/dL) | 8 | 53.5 | - | 55.9 | 55.3 | 2.13 | 0.91 |
| Cholesterol | (mg/dL) | 8 | 139.3 | 114 | 173 | - | 11.6 | 0.05 |
| Creatinine | (mg/dL) | 8 | 1.36^ab^ | 1.97^a^ | 0.89^b^ | - | 0.19 | 0.01 |

Different superscript alphabets of means in a row are significant differences at *P*-value≤0.05. SEM, standard error of the mean; BUN, blood urea nitrogen; ALT, alanine transaminase.

**Table S12 A systematically organized compilation of legislative documents from the European Parliament and the Council (EC) regarding insect production for food and feed, arranged chronologically.**

| **Date for announced/released** | **Document's title** | | | | | **Committee/art.** | **Amending regulation** | **Repealing regulation** | **Reference no.** |
| --- | --- | --- | --- | --- | --- | --- | --- | --- | --- |
| 22.05.2001 | Laying down rules for the prevention, control and eradication of certain transmissible spongiform encephalopathies. | | | | | (EC) No. 999/2001 | - | - | (1) |
|  |  |  |  |  |  |  |  |  |  |
|  |  |  |  |  |  |  |  |  |  |
| 28.01.2002 | Laying down the general principles and requirements of food law, establishing the European Food Safety Authority and laying down procedures in matters of food safety. | | | | | (EC) No. 178/2002 | - | - | (2) |
|  |  |  |  |  |  |  |  |  |  |
|  |  |  |  |  |  |  |  |  |  |
|  |  |  |  |  |  |  |  |  |  |
| 07.05.2002 | Undesirable substances in animal feed—Council statement | | | | | (EC) No. 32/2002 | - | - | (3) |
|  |  |  |  |  |  |  |  |  |  |
| 22.09.2003 | Additives for use in animal nutrition | | |  |  | (EC) No. 1831/2003 | - | - | (4) |
|  |  |  |  |  |  |  |  |  |  |
|  |  |  |  |  |  |  |  |  |  |
| 29.04.2004 | Official controls performed to ensure the verification of compliance with feed and food law, animal health and animal welfare rules | | | | | (EC) No. 882/2004 | - | - | (5) |
|  |  |  |  |  |  |  |  |  |  |
|  |  |  |  |  |  |  |  |  |  |
|  |  |  |  |  |  |  |  |  |  |
| 29.04.2004 | Laying down specific hygiene rules for food of animal origin | | | | | (EC) No. 853/2004 | - | - | (6) |
|  |  |  |  |  |  |  |  |  |  |
| 29.04.2004 | Laying down specific rules for the organization of official controls on products of animal origin intended for human consumption | | | | | (EC) No. 854/2004 | - | - | (7) |
|  |  |  |  |  |  |  |  |  |  |
|  |  |  |  |  |  |  |  |  |  |
|  |  |  |  |  |  |  |  |  |  |
| 15.11.2005 | Microbiological criteria for food stuffs | | | |  | (EC) No. 2073/2005 | - | - | (8) |
|  |  |  |  |  |  |  |  |  |  |
|  |  |  |  |  |  |  |  |  |  |
| 13.07.2009 | Placing on the market and use of feed | | |  |  | (EC) No. 767/2009 | (EC) No. 1831/2003 | Council Directive 79/373/EEC; Commission Directive 80/511/EEC, Council Directives 82/471/EEC, 83/228/EEC, 93/74/EEC, 93/113/EC and 96/25/EC and Commission Decision 2004/217/EC. | (9) |
|  |  |  |  |  |  |  |  |  |  |
|  |  |  |  |  |  |  |  |  |  |
|  |  |  |  |  |  |  |  |  |  |
|  |  |  |  |  |  |  |  |  |  |
|  |  |  |  |  |  |  |  |  |  |
| 21.10.2009 | Laying down health rules as regards animal by-products and derived products not intended for human consumption | | | | | (EC) No. 1069/2009 | - | (EC) No. 1774/2002 | (10) |
|  |  |  |  |  |  |  |  |  |  |
|  |  |  |  |  |  |  |  |  |  |
| 25.02.2011 | Laying down health rules as regards animal by-products and derived products not intended for human consumption | | | | | (EC) No. 142/2011 | - | - | (11) |
|  |  |  |  |  |  |  |  |  |  |
|  |  |  |  |  |  |  |  |  |  |
| 25.10.2011 | Provision of food information to consumers | | | |  | (EC) No. 1169/2011 | (EC) No. 1924/2006 and (EC) No. 1925/2006 | Commission Directive 87/250/EEC, Council Directive 90/496/EEC, Commission Directive 1999/10/EC, Directive 2000/13/EC of the European Parliament and of the Council, Commission Directives 2002/67/EC and 2008/5/EC and Commission Regulation (EC) No. 608/2004. | (12) |
|  |  |  |  |  |  |  |  |  |  |
|  |  |  |  |  |  |  |  |  |  |
|  |  |  |  |  |  |  |  |  |  |
|  |  |  |  |  |  |  |  |  |  |
|  |  |  |  |  |  |  |  |  |  |
|  |  |  |  |  |  |  |  |  |  |
|  |  |  |  |  |  |  |  |  |  |
| 25.11.2015 | Novel foods | |  |  |  | (EC) No. 2283/2015 | (EU) No. 1169/2011 | (EC) No. 258/97; (EC) No. 1852/2001 | (13) |
|  |  |  |  |  |  |  |  |  |  |
|  |  |  |  |  |  |  |  |  |  |
| 24.05.2017 | Provisions on processed animal protein | | | |  | (EC) No. 893/2017 | (EC) No. 999/2001; (EU) No. 142/2011 | - | (14) |
|  |  |  |  |  |  |  |  |  |  |
|  |  |  |  |  |  |  |  |  |  |
| 20.12.2017 | Laying down administrative and scientific requirements for applications | | | | | (EC) No. 2469/2017 | - | - | (15) |
|  |  |  |  |  |  |  |  |  |  |
|  |  |  |  |  |  |  |  |  |  |
| 17.08.2021 | Prohibition to feed nonruminant farmed animals, other than fur animals, with protein derived from animals | | | | | (EC) No. 1372/2021 | (EC) No. 999/2001 | - | (16) |
|  |  |  |  |  |  |  |  |  |  |

References

1. Commission Regulation (EC) No. 999/2001, Regulation (EC) No. 999/2001 of the European Parliament and of the Council of 22 May 2001 Laying Down Rules for the Prevention, Control and Eradication of Certain Transmissible Spongiform Encephalopathies; European Union: Luxemburg, 2001.
2. Regulation (EC) No. 178/2002 of the European Parliament and of the Council of 28 January 2002 Laying Down the General Principles and Requirements of Food Law, Establishing the European Food Safety Authority and Laying Down Procedures in Matters of Food Safety; European Union: Luxemburg, 2002.
3. Directive 2002/32/EC of the European Parliament and of the Council of 7 May 2002 on Undesirable Substances in Animal Feed—Council statement; European Union: Luxemburg, 2002.
4. Regulation (EC) No. 1831/2003 of the European Parliament and of the Council of 22 September 2003 on Additives for Use in Animal Nutrition; European Union: Luxemburg, 2003.
5. Regulation (EC) No. 882/2004 of the European Parliament and of the Council of 29 April 2004 on Official Controls Performed to Ensure the Verification of Compliance with Feed and Food Law, Animal Health and Animal Welfare Rules; European Union: Luxemburg, 2004.
6. Regulation (EC) No. 853/2004 of the European Parliament and of the Council of 29 April 2004 Laying Down Specific Hygiene Rules for Food of Animal Origin; European Union: Luxemburg, 2004.
7. Regulation (EC) No. 854/2004 of the European Parliament and of the Council of 29 April 2004 Laying Down Specific Rules for the Organisation of Official Controls on Products of Animal Origin Intended for Human Consumption; European Union: Luxemburg, 2004.
8. Commission Regulation (EC) No. 2073/2005 of 15 November 2005 on Microbiological Criteria for Foodstuffs; European Union: Luxemburg, 2005.
9. Regulation (EC) No. 767/2009, Regulation (EC) No. 767/2009 of the European Parliament and of the Council of 13 July 2009 on the Placing on the Market and Use of Feed, Amending European Parliament and Council Regulation (EC) No. 1831/2003 and Repealing Council Directive 79/373/EEC, Commission Directive 80/511/EEC, Council Directives 82/471/EEC, 83/228/EEC, 93/74/EEC, 93/113/EC and 96/25/EC and Commission Decision 2004/217/EC; European Union: Luxemburg, 2004.
10. Commission Regulation (EC) No. 1069/2009, Regulation (EC) No. 1069/2009 of the European Parliament and of the Council of 21 October 2009 Laying Down Health Rules as Regards Animal by-Products and Derived Products not Intended for Human Consumption and Repealing Regulation (EC) No. 1774/2002; European Union: Luxemburg, 2002.
11. Commission Regulation (EU) No. 142/2011, Commission Regulation (EU) No. 142/2011 of 25 February 2011 Implementing Regulation (EC) No. 1069/2009 of the European Parliament and of the Council Laying Down Health Rules as Regards Animal by-Products and Derived products not Intended for Human Consumption and Implementing Council Directive 97/78/EC as Regards Certain Samples and Items Exempt from Veterinary Checks at the Border under that Directive; European Union: Luxemburg, 2011.
12. Regulation (EU) No. 1169/2011 of the European Parliament and of the Council of 25 October 2011 on the Provision of Food Information to Consumers, Amending Regulations (EC) No. 1924/2006 and (EC) No. 1925/2006 of the European Parliament and of the Council, and Repealing Commission Directive 87/250/EEC, Council Directive 90/496/EEC, Commission Directive 1999/10/EC, Directive 2000/13/EC of the European Parliament and of the Council, Commission Directives 2002/67/EC and 2008/5/EC and Commission Regulation (EC) No. 608/2004; European Union: Luxemburg, 2004.
13. Regulation (EU) 2015/2283 of the European Parliament and of the Council of 25 November 2015 on Novel Foods, Amending Regulation (EU) No. 1169/2011 of the European Parliament and of the Council and Repealing Regulation (EC) No. 258/97 of the European Parliament and of the Council and Commission Regulation (EC) No. 1852/2001; European Union: Luxemburg, 2001.
14. Commission Regulation (EU) 2017/893 of 24 May 2017 Amending Annexes I and IV to Regulation (EC) No. 999/2001 of the European Parliament and of the Council and Annexes X, XIV and XV to Commission Regulation (EU) No. 142/2011 as Regards the Provisions on Processed Animal Protein; European Union: Luxemburg, 2017.
15. Commission Implementing Regulation (EU) 2017/2469 of 20 December 2017 Laying Down Administrative and Scientific Requirements for Applications Referred to in Article 10 of Regulation (EU) 2015/2283 of the European Parliament and of the Council on Novel Foods; European Union: Luxemburg, 2017.
16. Commission Regulation (EU) 2021/1372 of 17 August 2021 Amending Annex IV to Regulation (EC) No. 999/2001 of the European Parliament and of the Council as Regards the Prohibition to Feed Non-Ruminant Farmed Animals, Other than Fur Animals, with Protein Derived from Animals; European Union: Luxemburg, 2021.
