# Supplementary material for: Cutting-edge exploration of insect utilization in ruminant nutrition—feature and future: a systematic review and meta-analysis
Source: Front Vet Sci. 2024 Nov 20;11:1484870. doi: 10.3389/fvets.2024.1484870 (PMC11616318; doi:10.3389/fvets.2024.1484870)
Supplement: SUPPLEMENTARY MATERIAL 3 — References. [file Supplementary_file_3.docx]

**References**

1. Ahmed, E., Fukuma, N., Hanada, M., Nishida, T. (2021). Insects as novel ruminant feed and a potential mitigation strategy for methane emissions. Animals. 11(9):2648. <https://doi.org/10.3390/ani11092648>.
2. Ahmed, E., and Nishida, T. (2023). Optimal inclusion levels of cricket and silkworm as alternative ruminant feed: a study on their impacts on rumen fermentation and gas production. Sustainability. 15(2):1415. <https://doi.org/10.3390/su15021415>.
3. Akande, A.O., Jolayemi, O.S., Adelugba, V.A., Akande, S.T. (2020). Silkworm pupae (Bombyx mori) and locusts as alternative protein sources for high-energy biscuits. J Asia Pac Entomol. 23(1):234-41. <https://doi.org/10.1016/j.aspen.2020.01.003>.
4. Banday, M.T., Adil, S., Sheikh, I.U., Hamadani, H., Qadri, F.I., Sahfi, M.E., et al. (2023). The use of silkworm pupae (Bombyx mori) meal as an alternative protein source for poultry. J World's Poult Sci. 79(1):119-34. <https://doi.org/10.1080/00439339.2023.2163955>.
5. Bovera, F., Loponte, R., Marono, S., Piccolo, G., Parisi, G., Iaconisi, V., et al. (2016). Use of Tenebrio molitor larvae meal as protein source in broiler diet: Effect on growth performance, nutrient digestibility, and carcass and meat traits. J Anim Sci. 94(2):639-47. <https://doi.org/10.2527/jas.2015-9201>.
6. Chieco, C., Morrone, L., Bertazza, G., Cappellozza, S., Saviane, A., Gai, F., et al. (2019). The effect of strain and rearing medium on the chemical composition, fatty acid profile and carotenoid content in silkworm (Bombyx mori) pupae. Animals. 9(3):103. <https://doi.org/10.3390/ani9030103>.
7. Crosbie, M., Zhu, C., Shoveller, A.K., Huber, L.A. (2020). Standardized ileal digestible amino acids and net energy contents in full fat and defatted black soldier fly larvae meals (Hermetia illucens) fed to growing pigs. Transl anim sci. 4(3):txaa104. <https://doi.org/10.1093/tas/txaa104>.
8. David-Birman, T., Moshe, H., Lesmes, U. (2019). Impact of thermal processing on physicochemical properties of silk moth pupae (Bombyx mori) flour and in-vitro gastrointestinal proteolysis in adults and seniors. Food Res Int. 123:11-9. <https://doi.org/10.1016/j.foodres.2019.04.042>.
9. Daszkiewicz, T., Murawska, D., Kubiak, D., Han, J. (2022). Chemical composition and fatty acid profile of the pectoralis major muscle in broiler chickens fed diets with full-fat black soldier fly (Hermetia illucens) larvae meal. Animals. 12(4):464. <https://doi.org/10.3390/ani12040464>.
10. De Marco, M., Martínez, S., Hernandez, F., Madrid, J., Gai, F., Rotolo, L., et al. (2015). Nutritional value of two insect larval meals (Tenebrio molitor and Hermetia illucens) for broiler chickens: Apparent nutrient digestibility, apparent ileal amino acid digestibility and apparent metabolizable energy. Anim Feed Sci Technol. 209:211-8. <https://doi.org/10.1016/j.anifeedsci.2015.08.006>.
11. De Souza Vilela, J., Alvarenga, T.I., Andrew, N.R., McPhee, M., Kolakshyapati, M., Hopkins, D.L., Ruhnke I. (2021). Technological quality, amino acid and fatty acid profile of broiler meat enhanced by dietary inclusion of black soldier fly larvae. Foods. 10(2):297. <https://doi.org/10.3390/foods10020297>.
12. Ewald, N., Vidakovic, A., Langeland, M., Kiessling, A., Sampels, S., Lalander, C. (2020). Fatty acid composition of black soldier fly larvae (Hermetia illucens)–Possibilities and limitations for modification through diet. Waste Manag. 102:40-7. <https://doi.org/10.1016/j.wasman.2019.10.014>.
13. Fialho, A.T., Silva, A.S., Brito, C.O., Vale, P.A., Oliveira, C.J., Ribeiro, V. (2021). Nutritional composition of larvae of mealworm (Tenebrio molitor L.) and crickets (Gryllus assimilis) with potential usage in feed. Arquivo Brasileiro de Medicina Veterinária e Zootecnia. 73(2):539-42. <https://doi.org/10.1590/1678-4162-12158>.
14. Forkwa, T.F., John, K., Monica, A., Chrysantus, M.T., Jozef, V.B., Mik, V.D. (2021). Affordable Processing of Edible Orthopterans Provides a Highly Nutritive Source of Food Ingredients. Foods. 10(1):144. <https://doi.org/10.3390/foods10010144>.
15. Ghosh, S., Lee, S.M., Jung, C., Meyer-Rochow, V.B. (2017). Nutritional composition of five commercial edible insects in South Korea. J Asia Pac Entomol. 20(2):686-94. <https://doi.org/10.1016/j.aspen.2017.04.003>.
16. Giannetto, A., Oliva, S., Lanes, C.F., de Araújo Pedron, F., Savastano, D., Baviera, C., et al. (2020). Hermetia illucens (Diptera: Stratiomydae) larvae and prepupae: Biomass production, fatty acid profile and expression of key genes involved in lipid metabolism. J Biotechnol. 307:44-54. <https://doi.org/10.1016/j.jbiotec.2019.10.015>.
17. Hăbeanu, M., Gheorghe, A., Mihalcea, T. (2023). Nutritional value of silkworm pupae (Bombyx mori) with emphases on fatty acids profile and their potential applications for humans and animals. Insects. 14(3):254. <https://doi.org/10.3390/insects14030254>.
18. Hu, B., Li, C., Zhang, Z., Zhao, Q., Zhu, Y., Su, Z., Chen, Y. (2017). Microwave-assisted extraction of silkworm pupal oil and evaluation of its fatty acid composition, physicochemical properties and antioxidant activities. Food Chem. 231:348-55. <https://doi.org/10.1016/j.foodchem.2017.03.152>.
19. Khanal, P., Pandey, D., Næss, G., Cabrita, A.R., Fonseca, A.J., Maia, M.R., et al. (2023). Yellow mealworms (Tenebrio molitor) as an alternative animal feed source: A comprehensive characterization of nutritional values and the larval gut microbiome. J Clean Prod. 389:136104. <https://doi.org/10.1016/j.jclepro.2023.136104>.
20. Kim, S.K., Weaver, C.M., Choi, M.K. (2017). Proximate composition and mineral content of five edible insects consumed in Korea. CYTA - J Food. 15(1):143-6. <https://doi.org/10.1080/19476337.2016.1223172>.
21. Köhler, R., Kariuki, L., Lambert, C., Biesalski, H.K. (2019). Protein, amino acid and mineral composition of some edible insects from Thailand. J Asia Pac Entomol. 22(1):372-8. <https://doi.org/10.1016/j.aspen.2019.02.002>.
22. Kröncke, N., Neumeister, M., Benning, R. (2023). Near-infrared reflectance spectroscopy for quantitative analysis of fat and fatty acid content in living Tenebrio molitor larvae to detect the influence of substrate on larval composition. Insects. 14(2):114. <https://doi.org/10.3390/insects14020114>.
23. Kuntadi, K., Adalina, Y., Maharani, K.E. (2018). Nutritional compositions of six edible insects in Java. Indones J For Res. 5(1):57-68. <https://doi.org/10.20886/ijfr.2018.5.1.57-68>.
24. Kwon, M.G., Kim, D.S., Lee, J.H., Park, S.W., Choo, Y.K., Han, Y.S., et al. (2012). Isolation and analysis of natural compounds from silkworm pupae and effect of its extracts on alcohol detoxification. Entomol Res. 42(1):55-62. <https://doi.org/10.1111/j.1748-5967.2011.00439.x>.
25. Li, X., Dong, Y., Sun, Q., Tan, X., You, C., Huang, Y., Zhou, M. (2022). Growth and fatty acid composition of black soldier fly Hermetia illucens (Diptera: Stratiomyidae) larvae are influenced by dietary fat sources and levels. Animals. 12(4):486. <https://doi.org/10.3390/ani12040486>.
26. Liland, N.S., Biancarosa, I., Araujo, P., Biemans, D., Bruckner, C.G., Waagbø, R., et al. (2017). Modulation of nutrient composition of black soldier fly (Hermetia illucens) larvae by feeding seaweed-enriched media. PloS one. 12(8):e0183188. <https://doi.org/10.1371/journal.pone.0183188>.
27. Mancini, S., Mattioli, S., Paolucci, S., Fratini, F., Dal Bosco, A., Tuccinardi, T., Paci, G. (2021). Effect of cooking techniques on the in vitro protein digestibility, fatty acid profile, and oxidative status of mealworms (Tenebrio molitor). Front vet sci. 8:675572. <https://doi.org/10.3389/fvets.2021.675572>.
28. Marono, S., Piccolo, G., Loponte, R., Di Meo, C., Attia, Y.A., Nizza, A., Bovera, F. (2015). In vitro crude protein digestibility of Tenebrio molitor and Hermetia illucens insect meals and its correlation with chemical composition traits. Ital J Anim Sci. 14(3):3889. <https://doi.org/10.4081/ijas.2015.3889>.
29. Mlček, J., Adámková, A., Adámek, M., Borkovcová, M., Bednářová, M., Knížková, I. (2019). Fat from Tenebrionidae bugs–sterols content, fatty acid profiles, and cardiovascular risk indexes. Pol J Food Nutr Sci. 69(3):247-54. <https://doi.org/10.31883/pjfns/109666>.
30. Montalbán, A., Sánchez, C.J., Hernández, F., Schiavone, A., Madrid, J., Martínez-Miró, S. (2022). Effects of agro-industrial byproduct-based diets on the growth performance, digestibility, nutritional and microbiota composition of mealworm (Tenebrio molitor L.). Insects. 13(4):323. <https://doi.org/10.3390/insects13040323>.
31. Müller, A., Wolf, D., Gutzeit, H.O. (2017). The black soldier fly, Hermetia illucens–a promising source for sustainable production of proteins, lipids and bioactive substances. Zeitschrift für Naturforschung C. 72(9-10):351-63. <https://doi.org/10.1515/znc-2017-0030>.
32. Murugu, D.K., Onyango, A.N., Ndiritu, A.K., Osuga, I.M., Xavier, C., Nakimbugwe, D., Tanga, C.M. (2021). From farm to fork: crickets as alternative source of protein, minerals, and vitamins. Front nutr. 8:704002. <https://doi.org/10.3389/fnut.2021.704002>.
33. Nayohan, S., Susanto, I., Permata, D., Pangesti, R.T., Rahmadani, M., Jayanegara, A. (2022). Effect of dietary inclusion of black soldier fly larvae (Hermetia illucens) on broiler performance: A meta-analysis. E3S Web Conf. 335:00013. <https://doi.org/10.1051/e3sconf/202233500013>.
34. Onsongo, V.O., Osuga, I.M., Gachuiri, C.K., Wachira, A.M., Miano, D.M., Tanga, C.M., et al. (2018). Insects for income generation through animal feed: effect of dietary replacement of soybean and fish meal with black soldier fly meal on broiler growth and economic performance. J Econ Entomol. 111(4):1966-73. <https://doi.org/10.1093/jee/toy118>.
35. Orkusz, A., Dymińska, L., Banaś, K., Harasym, J. (2023). Chemical and Nutritional Fat Profile of Acheta domesticus, Gryllus bimaculatus, Tenebrio molitor and Rhynchophorus ferrugineus. Foods. 13(1):32. <https://doi.org/10.3390/foods13010032>.
36. Pereira, N.R., Ferrarese-Filho, O., Matsushita, M., de Souza, N.E. (2003). Proximate composition and fatty acid profile of Bombyx mori L. chrysalis toast. J Food Compos Anal. 16(4):451-7. <https://doi.org/10.1016/S0889-1575(03)00016-4>.
37. Phesatcha, B., Phesatcha, K., Viennaxay, B., Matra, M., Totakul, P., Wanapat, M. (2022). Cricket meal (Gryllus bimaculatus) as a protein supplement on in vitro fermentation characteristics and methane mitigation. Insects. 13(2):129. <https://doi.org/10.3390/insects13020129>.
38. Rabani, V., Cheatsazan, H., Davani, S. (2019). Proteomics and lipidomics of black soldier fly (Diptera: Stratiomyidae) and blow fly (Diptera: Calliphoridae) larvae. J Insect Sci. 19(3):29. <https://doi.org/10.1093/jisesa/iez050>.
39. Ravzanaadii, N., Kim, S.H., Choi, W.H., Hong, S.J., Kim, N.J. (2012). Nutritional value of mealworm, Tenebrio molitor as food source. Int J Indust Entomol. 25(1):93-8. <https://doi.org/10.7852/ijie.2012.25.1.093>.
40. Rawski, M., Mazurkiewicz, J., Kierończyk, B., Józefiak, D. (2020). Black soldier fly full-fat larvae meal as an alternative to fish meal and fish oil in Siberian sturgeon nutrition: The effects on physical properties of the feed, animal growth performance, and feed acceptance and utilization. Animals. 10(11):2119. <https://doi.org/10.3390/ani10112119>.
41. Razali, S.M. (2022). Physicochemical characteristics and microbiological quality of silkworm (Bombyx mori) larval and pupae powder: comparative study. Sains Malays. 51(2):547-58. <http://doi.org/10.17576/jsm-2022-5102-18>.
42. Renna, M., Coppa, M., Lussiana, C., Le Morvan, A., Gasco, L., Maxin, G. (2022). Full-fat insect meals in ruminant nutrition: in vitro rumen fermentation characteristics and lipid biohydrogenation. J Anim Sci Biotechnol. 13(1):138. <https://doi.org/10.1186/s40104-022-00792-2>.
43. Rodríguez-Ortega, A., Pino-Moreno, J.M., Ángeles-Campos, S.C., García-Pérez, Á., Barrón-Yánez, R.M., Callejas-Hernández, J. (2016). Valor nutritivo de larvas y pupas de gusano de seda (Bombyx mori)(Lepidoptera: Bombycidae). Revista Colombiana de Entomología. 42(1):69-74. <https://doi.org/10.25100/socolen.v42i1.6672>.
44. Romano, N., Fischer, H., Kumar, V., Francis, S.A., Sinha, A.K. (2022). Productivity, conversion ability, and biochemical composition of black soldier fly (Hermetia illucens) larvae fed with sweet potato, spent coffee or dough. Int J Trop Insect Sci. 42:183–190. <https://doi.org/10.1007/s42690-021-00532-5>.
45. Selaledi, L., and Mabelebele, M. (2021). The influence of drying methods on the chemical composition and body color of yellow mealworm (Tenebrio molitor L.). Insects. 12(4):333. <https://doi.org/10.3390/insects12040333>.
46. Shah, A.A., Totakul, P., Matra, M., Cherdthong, A., Hanboonsong, Y., Wanapat, M. (2022). Nutritional composition of various insects and potential uses as alternative protein sources in animal diets. Anim Biosci. 35(2):317. <https://doi.org/10.5713/ab.21.0447>.
47. Shumo, M., Osuga, I.M., Khamis, F.M., Tanga, C.M., Fiaboe, K.K., Subramanian, S., et al. (2019). The nutritive value of black soldier fly larvae reared on common organic waste streams in Kenya. Sci Rep. 9(1):10110. <https://doi.org/10.1038/s41598-019-46603-z>.
48. Smets, R., Verbinnen, B., Van De Voorde, I., Aerts, G., Claes, J., Van Der Borght, M. (2020). Sequential extraction and characterisation of lipids, proteins, and chitin from black soldier fly (Hermetia illucens) larvae, prepupae, and pupae. Waste Biomass Valor. 2020;11:6455-66. <https://doi.org/10.1007/s12649-019-00924-2>.
49. Spranghers, T., Ottoboni, M., Klootwijk, C., Ovyn, A., Deboosere, S., De Meulenaer, B., et al. (2017). Nutritional composition of black soldier fly (Hermetia illucens) prepupae reared on different organic waste substrates. J Sci Food Agric. 97(8):2594-600. <https://doi.org/10.1002/jsfa.8081>.
50. Taufek, N.M., Muin, H., Raji, A.A., Razak, S.A., Yusof, H.M., Alias, Z. (2016). Apparent digestibility coefficients and amino acid availability of cricket meal, Gryllus bimaculatus, and fishmeal in African catfish, Clarias gariepinus, diet. J World Aquac Soc. 47(6):798-805. <https://doi.org/10.1111/jwas.12302>.
51. Tomotake, H., Katagiri, M., Yamato, M. (2010). Silkworm pupae (Bombyx mori) are new sources of high quality protein and lipid. J Nutr Sci Vitaminol. 56(6):446-8. <https://doi.org/10.3177/jnsv.56.446>.
52. Tong, L., Yu, X., Liu, H. (2011). Insect food for astronauts: gas exchange in silkworms fed on mulberry and lettuce and the nutritional value of these insects for human consumption during deep space flights. Bull Entom Res. 101(5):613-22. <https://doi.org/10.1017/S0007485311000228>.
53. Tyshko, N.V., Zhminchenko, V.M., Nikitin, N.S., Trebukh, M.D., Shestakova, S.I., Pashorina, V.A., Sadykova, E.O. (2021). The comprehensive studies of Hermetia illucens larvae protein’s biological value. Probl Nutr. 90:49-58. <https://doi.org/10.33029/0042-8833-2021-90-5-49-58>.
54. Udomsil, N., Imsoonthornruksa, S., Gosalawit, C., Ketudat-Cairns, M. (2019). Nutritional values and functional properties of house cricket (Acheta domesticus) and field cricket (Gryllus bimaculatus). Food Sci Technol Res. 25(4):597-605. <https://doi.org/10.3136/fstr.25.597>.
55. Wei, Z.J., Liao, A.M., Zhang, H.X., Liu, J., Jiang, S.T. (2009). Optimization of supercritical carbon dioxide extraction of silkworm pupal oil applying the response surface methodology. Bioresour Technol. 100(18):4214-9. <https://doi.org/10.1016/j.biortech.2009.04.010>.
56. Wu, R.A., Ding, Q., Yin, L., Chi, X., Sun, N., He, R., et al. (2020). Comparison of the nutritional value of mysore thorn borer (Anoplophora chinensis) and mealworm larva (Tenebrio molitor): Amino acid, fatty acid, and element profiles. Food Chem. 323:126818. <https://doi.org/10.1016/j.foodchem.2020.126818>.
57. Yu, X., He, Q., Wang, D. (2021). Dynamic analysis of major components in the different developmental stages of Tenebrio molitor. Front nutr. 8:689746. https://doi.org/10.3389/fnut.2021.689746.
